# The role of pharmacists and pharmacy team members in the prevention, preparedness, response and recovery of outbreaks – a global rapid systematic review

**DOI:** 10.1186/s12889-026-27937-6

**Published:** 2026-06-05

**Authors:** Rachel Berry, Anna Wilkinson, Aliya Turk, Bee Yean Ng, Sadie Pinkney, Bhavna Halai, Agatha Emoche, Tracey Thornley, Diane Ashiru-Oredope

**Affiliations:** 1 Antimicrobial Resistance and Healthcare Associated Infections Division, UK Health Security Agency, 10 South Colonnade, Canary Wharf, London, E14 4PU United Kingdom; 2 All Hazards Public Health Response Division, UK Health Security Agency, London, United Kingdom; 3 Chief Data Office, UK Health Security Agency, London, United Kingdom; 4 School of Pharmacy and Pharmaceutical Sciences, University of Liverpool, Liverpool, United Kingdom; 5 Medicines Optimisation and IR(ME)R, Care Quality Commission, London, United Kingdom; 6 School of Pharmacy, University of Nottingham, Nottingham, United Kingdom

**Keywords:** Pharmacy professional, Outbreak, Professional role, Prevention, Preparedness, Response, Recovery, Pharmaceutical public health, Pharmacoequity

## Abstract

**Background:**

Pharmacists and their teams can undertake pharmaceutical public health roles during outbreaks within the World Health Organization (WHO) emergency cycle of prevention, preparedness, response, and recovery. These may be at micro- (individual), meso- (regional or organisational) or macro- (national) levels.

The primary outcome was to identify pharmacy team members' roles in outbreaks (excluding COVID-19) and classify by WHO emergency cycle phase and intervention level.

**Methods:**

We conducted a rapid systematic review (PROSPERO: CRD42024617152) in Embase, Medline, and SCOPUS databases including articles published from 2014 to Feb 2025. After duplicate removal, titles/abstracts and full texts were screened, extracted and assessed for bias by one reviewer, with 10% second-checked. Thematic synthesis was used and described narratively to reflect variability in the studies.

**Results:**

From 161 articles across all WHO regions, 145 distinct thematically derived role categories were identified. Most were in the response phase (46%), followed by prevention (29%), preparedness (15%), and recovery (10%). Roles were primarily at meso-level (37%) and micro-level (35%), with fewer at macro-level (28%). 34% of the articles reported contributions related to health inequalities. Reported barriers were clustered into five themes: knowledge/training gaps, regulatory restrictions, resource and infrastructure constraints, communication, and cultural factors.

**Conclusion:**

Pharmacy team members have roles during non-COVID outbreaks across all stages of the WHO emergency cycle at all levels. However, their contributions are less well documented in recovery and at macro-level. Integrating pharmacy roles into emergency frameworks, supported by regulatory reform, funding, and training, may support health-system resilience, and reduce health inequalities.

**Supplementary Information:**

The online version contains supplementary material available at 10.1186/s12889-026-27937-6.

## Introduction

Pharmacists are trained healthcare professionals who ensure the responsible use of medicines across healthcare, including in sectors such as community, hospital, industrial, academic, and emergency services. They work collaboratively with other pharmacy team members involved in pharmaceutical service delivery, such as registered or regulated pharmacy technicians, assistants, trainees, students, and academic faculty, to contribute to the safety, quality, and efficiency of health systems [1].

The World Health Organization (WHO) defines a disease outbreak as the occurrence of disease cases exceeding normal expectancy and notes that outbreaks may be caused by infections, chemicals, or radioactive materials [2]. WHO outlines a comprehensive emergency management cycle consisting of four interconnected phases: prevention, preparedness, response, and recovery [3].

In many countries, pharmacists possess pharmaceutical public health skills that support their contribution to health protection activities (including those relating to outbreaks) at micro- (individual or patient-facing), meso- (regional or organisational) or macro- (national) levels [4].

- Micro-level: Direct patient-facing services like health promotion, screenings, and medication advice, and personal level activity such as self-education and maintaining competence.
- Meso-level: Organisational or community-level interventions such as health fairs, chronic disease programs, and collaboration with community groups, and organisational activities such as supporting personnel planning and education.
- Macro-level: Policy and system-level roles including national strategy development, commissioning services, and influencing public health policy, or activities undertaken at national level such as coordinating work or being part of national multi-disciplinary (MDT) teams.

International guidance focusing on emergency preparedness and pharmaceutical workforce development is available [5]. This emphasises the importance of integrating pharmacists and pharmacy teams into national outbreak response plans to strengthen resilience and improve health outcomes internationally.

The COVID‑19 pandemic elevated the visibility and scope of pharmacy roles in outbreak prevention and control, including evidence of expanded roles such as teleconsultations, home delivery of medications, enhanced patient education, and hygiene protocols [6] and contributions to antibiotic stewardship such as guiding rational antimicrobial use and combating misinformation [7]. Frameworks emphasize the importance of integrating pharmacists into all stages of outbreak management [8], but the available literature outside the COVID‑19 context remains fragmented and largely descriptive. Much of the existing evidence focuses on individual outbreaks or settings and has not been systematically synthesised to examine the role of pharmacy team members across different outbreaks, phases of the emergency cycle, and health‑system contexts [9]. This lack of consolidated evidence limits understanding of the scope, consistency, and impact of pharmacy team involvement beyond COVID-19 and highlights the need for a non-COVID synthesis of pharmacist and pharmacy team members roles during outbreaks.

This rapid systematic review aimed to identify pharmacists’ and pharmacy team members’ contributions to non-COVID outbreaks and to classify these roles according to the WHO emergency cycle [3] and level of intervention [4]. The secondary outcome was to identify any contributions to addressing health inequalities and barriers to these roles, services or activities.

## Method

This review followed the Preferred Reporting Items for Systematic Reviews and Meta-Analyses (PRISMA) guidelines [10] with adaptations for a rapid review due to a requirement for prompt information to support timely policy development and resource limitations as per published recommendations [11, 12]. These included restriction of the publication period to after 2014 to ensure current relevancy, limiting dual screening and extraction to 10% of included articles, and exclusion of grey literature and articles not accessible through institutional subscriptions of the project group. For the PRISMA checklist see Additional Information Table S1; recommendations by Stevens et al. [13] were also followed for reporting of a rapid systematic review. The protocol was registered with PROSPERO (CRD42024617152).

### Search strategy

A systematic literature search was conducted in Embase, MEDLINE and Scopus in February 2025. The search strategy was developed and refined by the authors (R.B., A.W., A.T., A.E., T.T., and D.A.O.) with advice from UK Health Security Agency Knowledge and Evidence specialists. The search focused on studies describing roles or services provided by pharmacists or pharmacy team members in outbreaks (as per the WHO definition [2], excluding COVID-19) in all settings.

Articles from all publication types (including peer reviewed articles, opinion pieces, letter, abstracts and posters for conferences) published between 2014 and February 2025 in any language describing roles of pharmacists, pharmacy technicians, pharmacy students or other pharmacy team members in non-COVID outbreaks (including pharmacy services/settings) were included. Articles discussing roles of non-pharmacy team members, staff or healthcare professionals, or solely discussing COVID 19 or outbreaks or epidemics not included within WHO definition e.g. obesity, opioid use were excluded.

A broad range of publication types was included in the searches as pharmacy roles are frequently not the primary focus of outbreak‑related publications; adopting more restrictive criteria would have risked omitting relevant roles reported within opinion pieces, letters, systematic reviews, and conference proceedings.

A broad range of pharmacy staff was included in the searches, reflecting the collaborative nature of pharmaceutical service delivery. Outbreak‑related roles are frequently reported at a team rather than individual professional level, therefore adopting narrower inclusion criteria would have risked omitting relevant contributions described within the published literature.

Opioid‑ and obesity‑related search terms were omitted from the final search strategy, as iterative searches demonstrated that these topics did not meet the review inclusion criteria as an outbreak as per the WHO definition, and consistently yielded a high volume of irrelevant articles.

A summary of the search strategy is shown in Table 1 (for detailed search strategy see Additional Information Table S2).

**Table 1:** Summary search strategy

Pharmacy professional	AND	Outbreaks	NOT	COVID or other
Pharmacists		Disease Outbreaks		covid*
Pharmacy technicians		Epidemics		corona*
Pharmacy staff		pandemic preparedness		obesity
Pharmacy students		biohazard release		opioid
pharmacy residencies		chemical hazard release		
clinical pharmacist		radioactive hazard release		
hospital pharmacist		HIV		
community pharmacist		STD		
pharmacies		Anthrax		
Community pharmacy services		influenza, human		
pharmacy service, hospital		Chikungunya		
pharmaceutical services, online		Cholera		
pharmaceutical services		Crimean-Congo haemorrhagic fever		
schools, pharmacy		Dengue		
pharmacy research		Diphtheria		
pharmacy practice		Ebola		
pharmacy		Hepatitis		
		Influenza		
		Measles		
		Meningitis		
		Middle East respiratory syndrome coronavirus		
		Plague		
		Poliomyelitis		
		Rift valley fever		
		Severe acute respiratory syndrome		
		Typhoid fever		
		Viral haemorrhagic fevers		
		Yellow fever		
		Zika virus		
		mpox		
		resistant infection		

### Screening

All identified articles were imported into Rayyan™ and duplicates were removed. Initially, all reviewers screened the first 10 records to refine inclusion criteria and ensure consistency. Thereafter, each article was screened by one of the reviewers (R.B., A.W., A.T., B.Y.N., S.P., and B.H.) with 10% reviewed by a second reviewer (10% randomly assigned by Rayyan™). Discrepancies were resolved by consensus through discussion or by a third reviewer (D.A.O) whose decision was final; full text was accessed if needed. Rayyan™ was used to track inclusion/exclusion decisions. Non‑English language articles were screened using automated translation tools.

### Data extraction

A standardised data extraction tool was designed with specified column headings using Microsoft Excel™. Each article was extracted by one of the reviewers (R.B., A.W., A.T., B.Y.N., S.P., and B.H.) and 10% reviewed in duplicate (every 10th article when alphabetical by author). All discrepancies were resolved by consensus through discussion. Non‑English language articles were translated using machine‑assisted translation, with reviewer verification during screening and data extraction to ensure accuracy where necessary.

Extracted data included:

- Study characteristics (country, outbreak type, pharmacy team member discussed, setting).
- Roles identified.
- Any discussion relating to health inequalities or barriers to role delivery.

### Risk of bias assessment

Due to the wide range of articles included in the rapid review, assessment of risk of bias was completed using multiple checklist tools. These were the Mixed Methods Appraisal Tool (MMAT) [14] for qualitative studies, randomised controlled trials, non-randomised studies, quantitative descriptive studies and mixed-method studies, the JBI checklist for Case Reports [15] for case reports, the JBI checklist for Narrative, Expert Opinion, Policy/Consensus Guidance [16] for letters, opinions, narrative reports and policy or consensus guidance, and the JBI checklist for Systematic Reviews [17] for systematic reviews. Each article was reviewed by one reviewer (R.B., A.W., A.T., B.Y.N., S.P., and B.H.) with 10% reviewed by a second (the 10th article when ordered alphabetically by author, starting at the fifth article). No discrepancies were identified. For non-English language articles, risk of bias analysis was undertaken using the translated texts, with reference back to the original articles in cases of ambiguity.

### Data synthesis

A narrative synthesis was conducted due to heterogeneity in article type, study design, setting and roles identified. The results were presented using narrative description, thematic analysis and descriptive statistics.

The primary aim was to collate roles of pharmacists or pharmacy team members in outbreaks (excluding COVID-19). Discrete role descriptions were extracted verbatim from each included source and initially coded to generate a broad set of roles. These were then reviewed and refined through iterative comparison across sources. Conceptually similar roles were combined where they related to the same underlying function, despite differences in wording or context, and where there was no meaningful difference in intent.

The roles were subsequently categorised by level (micro-, meso- or macro- level) and stage of the emergency cycle (preparedness, prevention, recovery or response) based on their primary focus as described in the source text. Where a role could theoretically apply to more than one stage or level, classification was based on the stage or level most clearly emphasised in the source text. In cases where a source explicitly described the same role operating across multiple stages or levels, it was recorded under each relevant category. Thematic coding was carried out by one lead author with clinical expertise (R.B.) with 10% independently double-coded by a second author (B.H.). Prior to formal coding, the two authors met to discuss the analytic approach and reviewed example data extracts to calibrate coding decisions. No discrepancies were identified between coders in the 10% that was checked. Where the primary coder expressed uncertainty regarding classification during analysis, these cases were discussed between the two authors and resolved through reference back to the original text.

Subgroup analyses were conducted to examine whether reported roles varied by country income level, pharmacy team member involved, and outbreak type. Country income level was determined through classification of the reported role taking place in a high-income country (HIC) or low/middle income country (LMIC) as defined by the 2025 World Bank definition [18] and articles with multiple countries were excluded to avoid misclassification across heterogeneous economic and healthcare contexts. Outbreak type was defined as being infectious disease or ‘other’. ‘Other’ included outbreaks related to food or water contamination, bioterrorism, chemical release, radiation release, or poisonings as per the WHO outbreak definition.

All thematic organisation, filtering, and mapping processes were carried out using Microsoft Excel™.

The secondary outcome was to identify any contributions to addressing health inequalities and barriers to these roles, services or activities; where these were discussed within a paper the key themes were extracted as verbatim and thematically coded and summarised narratively to provide an overview of key learning.

For non-English language articles, data coding, synthesis and sub-analysis was undertaken using translated texts, with reference back to the original articles in cases of ambiguity.

## Results

**Fig. 1:**
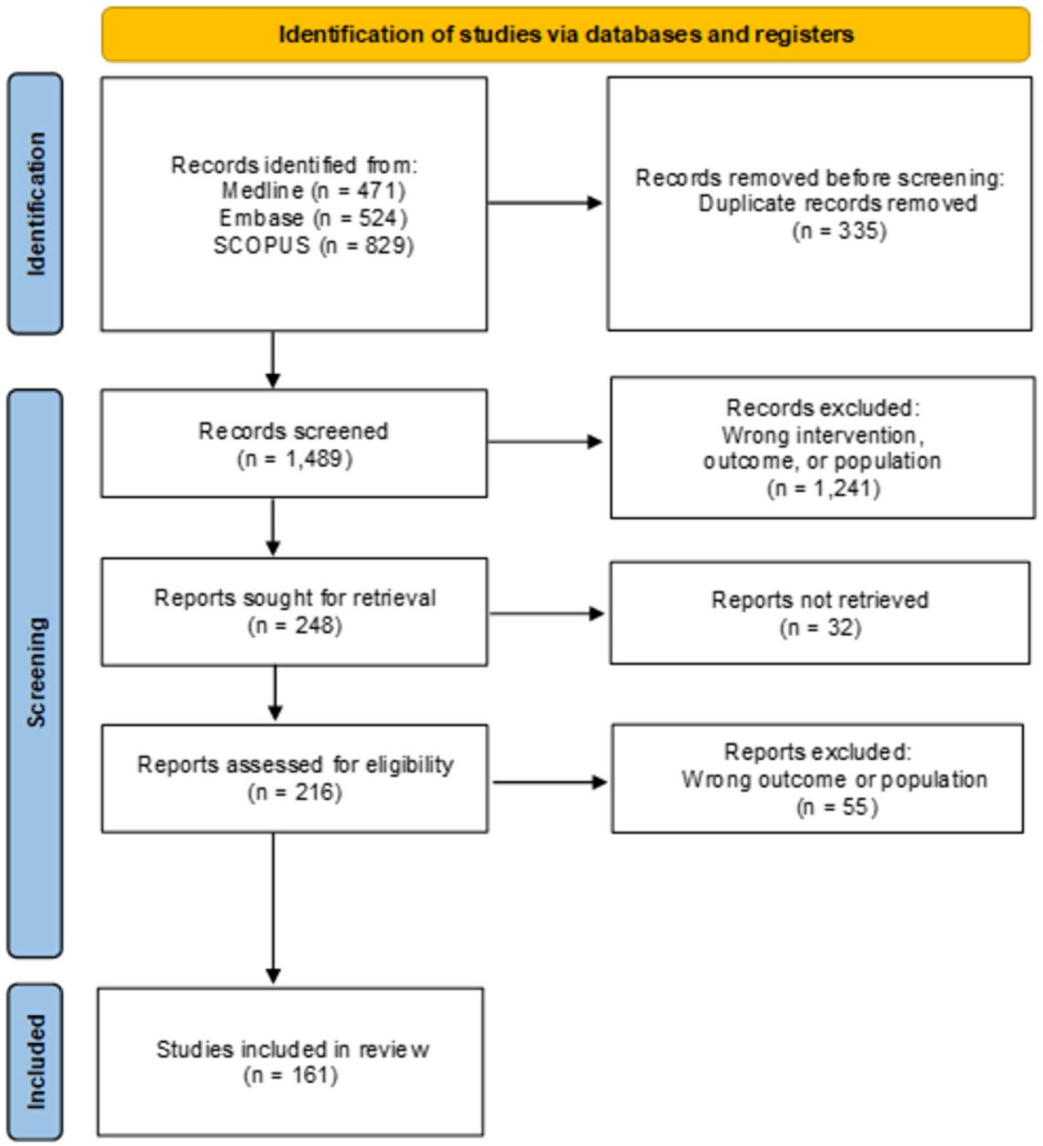
PRISMA flowchart of included articles

The searches returned 1,824 records. After removing duplicates, 1,489 papers were screened on title and abstract. Of these 1,241 were excluded leaving 248 for full article screening. Of these, 32 could not be retrieved (see Additional Information Table S3), and a further 55 were excluded because they met the exclusion criteria, leaving 161 eligible articles (Fig. 1).

### Characteristics of the identified articles

A summary of article characteristics is provided in Table 2. For the full extraction table see Additional Information Table S4.

**Table 2:** Summary of characteristics of included articles

Characteristics	Number of articles % (Total = 161)	References
World Health Organization Region		
Region of the Americas	60 (37.3%)	[19–78]
European Region	29 (18.0%)	[79–107]
Western Pacific	18 (11.2%)	[108–125]
Multiple countries included	18 (10.6%)	[126–143]
Eastern Mediterranean	17 (10.6%)	[144–160]
African Region	11 (7.5%)	[161–171]
South-East Asia	8 (5.0%)	[172–179]
Outbreak Type		
Not specific - pandemic, epidemic etc.	26 (16.1%)	[32, 33, 42, 54, 61, 75, 86, 87, 90, 94, 99, 104, 107, 109, 117, 128, 131, 132, 136, 139, 141, 143, 148, 160, 163, 172]
Influenza	23 (14.2%)	[21, 34, 39, 48, 63, 64, 70–72, 74, 79, 83, 88, 93, 100, 102, 111, 113, 115, 116, 118, 127, 154]
Not specific - infectious diseases	17 (10.6%)	[31, 37, 49, 52, 85, 89, 103, 105, 106, 108, 110, 112, 119, 129, 133, 162, 179]
Ebola virus	14 (8.7%)	[35, 43, 73, 78,82, 130, 134, 135, 140, 165–169]
Mpox or Monkeypox	14 (8.7%)	[28, 40, 57, 60, 123, 147, 149, 152, 153, 155–159]
Contamination of pharmaceutical product	13 (8.1%)	[19, 38, 47, 50, 53, 56, 59, 65, 68, 80, 97, 126, 171]
Human immunodeficiency virus (HIV)	12 (7.5%)	[22, 23, 26, 41, 51, 58, 69, 90, 92, 120, 164, 170]
Poisoning	4 (2.5%)	[36, 62, 84, 144]
Zika	4 (2.5%)	[25, 76, 77, 121]
Food or water contamination	3 (1.8%)	[81, 125, 146]
Measles	3 (1.8%)	[44, 46, 67]
Tuberculosis	3 (1.8%)	[122, 142, 178]
Biohazard	2 (1.2%)	[55, 96]
Clostridium difficile	2 (1.2%)	[45, 98]
Diphtheria	2 (1.2%)	[161, 177]
Human immunodeficiency virus (HIV) and Hepatitis C (HVC)	2 (1.2%)	[29, 30]
Acute hemorrhagic conjunctivitis	1 (0.6%)	[173]
Antimicrobial resistance (AMR)	1 (0.6%)	[151]
Cholera	1 (0.6%)	[176]
Crimean Congo Haemorrhagic Fever	1 (0.6%)	[150]
Dengue	1 (0.6%)	[175]
Enterovirus D68	1 (0.6%)	[27]
Headlice	1 (0.6%)	[137]
Impetigo	1 (0.6%)	[101]
Middle East Respiratory Syndrome	1 (0.6%)	[145]
Multi-drug resistant organism outbreak	1 (0.6%)	[114]
Mumps	1 (0.6%)	[20]
Paediatric diarrhoeal disease	1 (0.6%)	[124]
Pertussis	1 (0.6%)	[66]
Respiratory Syncytial Virus	1 (0.6%)	[138]
Rubella	1 (0.6%)	[174]
Scabies	1 (0.6%)	[24]
Smallpox	1 (0.6%)	[95]
Job Title		
Pharmacist	92 (57.1%)	[20, 24, 25, 27, 29–31, 33, 36, 39–42, 44–47, 51–57, 59, 61–64, 67, 69, 71, 72, 76–81, 84, 86, 88–91, 93, 95, 96, 98, 100, 101, 106–109, 117, 118, 120–122, 124, 129, 131, 132, 134, 136–139, 141, 142, 145–149, 153, 155, 157–160, 162, 164, 166–169, 174, 177–179]
Not specified	50 (31.1%)	[19, 21–23, 26, 35, 43, 48, 50, 58, 60, 65, 66, 68, 73, 74, 82, 83, 85, 87, 92, 94, 97, 99, 102–105, 110–116, 119, 126, 127, 130, 133, 140, 144, 154, 161, 170–173, 175, 176]
Multiple pharmacy team members	10 (6.2%)	[32, 34, 37, 38, 70, 75, 125, 128, 135, 151]
Pharmacy student	6 (3.7%)	[28, 49, 123, 150, 152, 156]
Pharmacy technician	1 (0.6%)	[163]
Pharmacy dispenser	1 (0.6%)	[165]
Pharmacy school faculty	1 (0.6%)	[143]
Setting		
Retail/ community	66 (41%)	[21–23, 26, 28–31, 34, 39, 46, 61, 63,64, 66, 67, 69–72, 75, 81–83, 86, 88, 90–95, 100–107, 109–112, 115, 117–122, 127, 131, 132, 137, 138, 141, 142, 147, 154, 160, 161, 164, 173, 176, 177]
Hospital	30 (18.6%)	[24, 32, 35, 36, 42–45, 54, 55, 57, 60, 62, 74, 78, 80, 97, 98, 114, 124, 129, 134, 140, 144, 145, 158, 166, 171, 172, 175]
Not specified	24 (14.9%)	[20, 25, 27, 33, 51, 58, 73, 79, 84, 89, 116, 128, 139, 146, 148, 149, 155–157, 162, 165, 168, 169, 179]
Multiple settings	12 (7.5%)	[19, 37, 76, 96, 99, 125, 135, 136, 151, 153, 159, 178]
Compounding pharmacy or centre	9 (5.6%)	[38, 47, 50, 53, 56, 59, 65, 68, 126]
Primary care	6 (3.7%)	[40, 77, 87, 163, 170, 174]
Higher Education Institutions	5 (3.1%)	[49, 123, 143, 150, 152]
Industry	3 (1.8%)	[113, 130, 133]
Long term care setting	2 (1.2%)	[48, 85]
Public health workforce	2 (1.2%)	[52, 108]
Prisons	1 (0.6%)	[41]
Research pharmacy	1 (0.6%)	[167]

Among the 161 included articles, 43 individual countries were discussed. The most frequently represented countries were the United States of America (57/161, 35.4%) [19–75], China (9/161, 5.6%) [108–116], Saudi Arabia (6/161, 3.7%) [144–149], France (5/161, 3.1%) [79–83], and Spain (5/161, 3.1%) [84–88]. 18 articles discussed multiple countries [126–143].

Most of the identified articles were quantitative descriptive studies (49/161, 30%), followed by narrative articles (24/161, 14.9%), expert opinion articles (23/161, 14.3%) and qualitative studies (19/161, 11.8%). Other types of articles, including policy and consensus guidance, case reports, non-randomised studies, systematic reviews, randomised control trials, conference abstracts and protocols had less than 10 articles each.

### Risk of bias assessment

Overall, the included studies were of moderate quality, as only 65 of the included studies (40.4%) fulfilled 100% of the relevant assessment criteria, 35 studies (21.7%) fulfilled between 75 and 99%, and 31 fulfilled between 50 and 74% (19.3%). A number fulfilled less than 50% (30/161, 18.6%) and ten studies were excluded from Risk of Bias assessment as they were conference abstracts or protocols. For full Risk of Bias assessment see Additional Information Table S5.

### Roles of pharmacy professionals

A total of 145 thematically derived role categories for pharmacists and pharmacy team members in non-COVID 19 outbreaks were identified (Table 3).

**Table 3:** Roles identified, by stage of cycle, level of intervention and country

Level	Micro				Meso				Macro			
Emergency Planning Cycle Stage		HIC	LMIC	Multiple countries		HIC	LMIC	Multiple countries		HIC	LMIC	Multiple countries
Preparedness	Knowledge of managing outbreaks			x	Provide education for pharmacy professionals in organisation	x		x	Development of legislation to support national emergency planning			x
	Undertake self-education	x	x	x	Business continuity planning to allow continuation of pharmacy services	x	x	x	Development of national emergency plans			x
	Maintain professional competence	x		x	Development of organisational emergency planning guidance and policies			x	Integration of pharmacies into national emergency planning	x		
	Knowledge of how to report key diseases		x		Development of organisational treatment policy or guidance	x		x	Advocate/lobby for better national public health measures			x
					Involvement in emergency planning at organisational level	x	x	x	Increase awareness of national opportunities for pharmacy professionals in public health roles	x		
					Take part in organisational preparedness simulations			x	Part of Multi-disciplinary Team that leads on national preparedness	x		
					Consider the use of novel solutions to provide usual pharmacy services			x	Encourage national partnerships with pharmaceutical companies			x
					Stock management and distribution of treatments, preventative measures or consumables – organisation	x			Disease surveillance - evaluation of national surveillance systems			x
					Disease surveillance - use of pharmacy medicines sale data for early outbreak detection, recognition and reporting of unusual medication requests	x	x	x				
Prevention	Knowledge of vaccination policy, guidance or recommendations		x		Development of vaccination policy, guidance or recommendations	x		x	Vaccine development and production - national	x		x
	Vaccination of patients	x	x	x	Vaccine production - organisational			x	Vaccine distribution		x	x
	Dispensing of vaccines	x	x	x	Vaccine clinical trials - organisational involvement		x	x	National education for pharmacy professionals about vaccination	x		x
	Assessing vaccination status	x			Vaccine distribution	x	x	x	National vaccination programme planning and implementation	x		
	Public education about vaccination	x	x	x	Organisational involvement in national vaccination program	x			Advocate/lobby for access to vaccination - sharing with other countries, unregistered patient access	x		x
	Patient education or counselling about vaccination, including reducing misinformation	x	x	x	Recognition and assessment of high-risk patients requiring vaccination - liaison with local schools	x			Quality assurance of new vaccines			x
	Recognition and assessment of high-risk patients requiring vaccination	x			Development of Infection, Prevention, and Control policy, guidance or recommendations			x	Development of Infection, Prevention, and Control policy, guidance or recommendations	x	x	
	Get recommended vaccinations			x	Follow Infection, Prevention, and Control policy, guidance or recommendations	x	x	x	National public health program planning and implementation	x		
	Knowledge of Infection, Prevention, and Control policy, guidance or recommendations	x	x		Support Infection, Prevention, and Control in care homes	x			Support national antimicrobial stewardship		x	
	Follow Infection, Prevention, and Control recommendations	x			Part of Infection, Prevention, and Control Multi-disciplinary Team	x		x	National education for Health Care Workers about antimicrobial stewardship			x
	Public education about prevention	x			Support organisational antimicrobial stewardship		x					
	Patient education or counselling about prevention	x	x	x	Production of preventative measures - alcohol based hand sanitiser		x	x				
	Support patient level antimicrobial stewardship	x	x		Educate Health Care Workers on how to produce preventative measures - alcohol based hand sanitiser		x					
	Educate Health Care Workers about antimicrobial stewardship			x	Development of aseptic policy or guidance for pharmaceutical products	x						
	Supply preventative medicines or measures to patients	x	x		Follow aseptic policy or guidance when making up pharmaceutical products	x		x				
	Preventative medicine adherence support	x	x									
	Follow aseptic policy or guidance when making up pharmaceutical products	x										
Response	Knowledge about the disease/outbreak	x	x		Team spirit			x	Leadership role at national level	x	x	x
	Knowledge about Health Technology Appraisals		x		Leadership role at organisational level	x		x	Support Health Care Worker and pharmacist education		x	
	Undertake self-education	x	x	x	Enable expanded job roles at organisational level	x			Advocate/lobby for patients		x	
	Undertake expanded job roles	x		x	Personnel management	x			Personnel management	x		
	Personal composure			x	Support organisational or regional outbreak management		x	x	Development of national treatment policy, recommendations or guidance	x	x	
	Respond to outbreak		x		Disease surveillance - use of pharmacy data	x	x		Develop, review or update legislation	x	x	x
	Disease surveillance - reporting of cases	x	x	x	Changes in ways of working to manage demand	x			Logistics and distribution of medicines or consumables		x	
	First point of patient contact	x	x	x	Development of organisational policy or guidance	x	x		Manage medicine and consumable supply issues		x	
	Provide First Aid		x		Maintain organisational compliance with legislation, policy, recommendation or guidance	x		x	Management of national drug costs		x	
	Symptom recognition and diagnosis	x	x	x	Stock management of medicines and consumables	x	x	x	Part of national Multi-disciplinary Team for outbreak response	x		
	Triage and appropriate referral of patients	x	x	x	Logistics and distribution of medicines or consumables	x	x	x	Collaboration with others working at national level	x	x	x
	Patient education and advice (including via digital platforms)	x	x	x	Manage medicine and consumable supply issues	x	x	x	Communication with others working at national level	x		x
	Point-of-care testing	x	x	x	Prevent hoarding or diversion of medicines			x	Develop national public education resources and campaigns		x	
	Pharmaceutical care of patients	x	x	x	Part of outbreak response Multi-Disciplinary Team	x	x	x	Pharmaceutical industry role in developing treatments		x	
	Supply of treatment to patients (Sales or dispensing)	x	x	x	Collaboration with others working at regional or organisational level	x		x	Advocate/lobby for research and clinical trials	x		
	Follow treatment guidance		x		Communication with others working at regional or organisational level	x		x	Clinical trials - design and implementation	x		
	Adherence support for treatment	x	x	x	Involvement in public health campaigns	x		x	Quality assurance of new products			x
	Maintain accurate and complete patient records.		x	x	Clinical trials - organisational involvement	x			Pharmaceutical product source identification	x		
	Involvement in patient identification, notification, and contract tracing	x			Research - organisational involvement		x					
	Pharmaceutical management of long-term conditions	x	x		Clinical trials - changes in ways of working	x						
	Continued supply of medicines for long-term conditions	x	x	x	Pharmaceutical product source identification	x						
	Stay within law for supply of medicines	x		x	Recall of contaminated pharmaceutical product	x	x					
	Manage supply issues and medicines shortages	x										
	Public education (including via digital platforms) to reduce misinformation panic and fear	x	x	x								
	Educate Health Care Workers	x	x									
	Contribute to clinical trials and research	x	x	x								
	Supply of investigational medicines to patients	x										
Recovery	Self-reflection			x	Post learning analysis and sharing	x	x	x	Review and update national protocol, policies, and guidelines			x
	Contribution to post learning analysis			x	Review and update organisational protocol, policies, and guidelines	x		x	Review legislation			x
	Long-term follow up and management of lasting effects of outbreak	x		x	Resumption of usual activity			x	Review partnership and joint working to inform future response			x
					Liaise with wider stakeholders regarding recovery			x	Collaborate on national reports about learning from outbreaks	x		
					Relocate personnel and equipment			x				
					Shut down temporary premises			x				
					Forecasting organisational recovery needs			x				

**Fig. 2:**
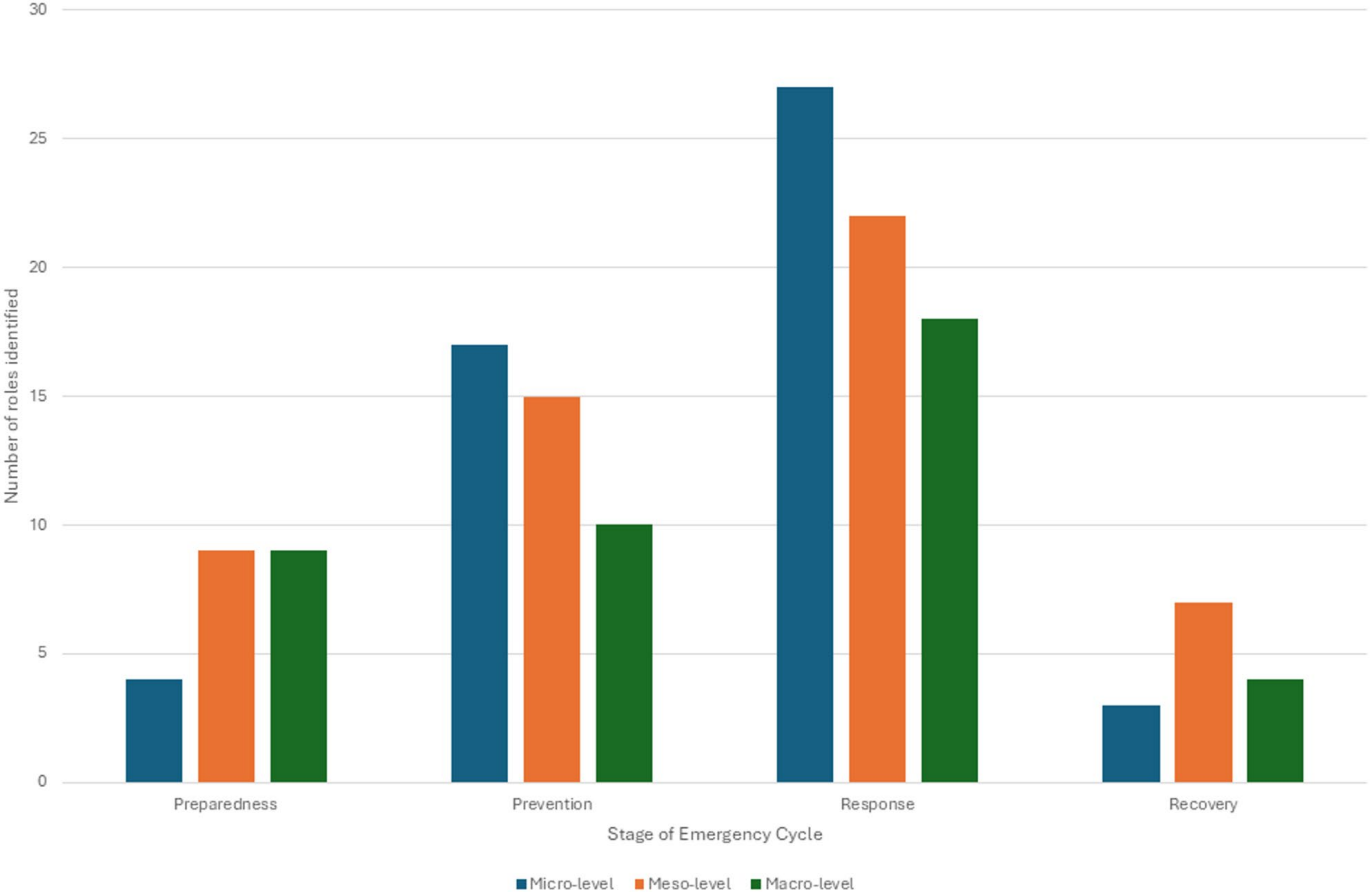
Roles by cycle stage and intervention level

These roles ranged across the stages of the WHO emergency cycle and the level of intervention (Fig. 2).

**Fig. 3:**
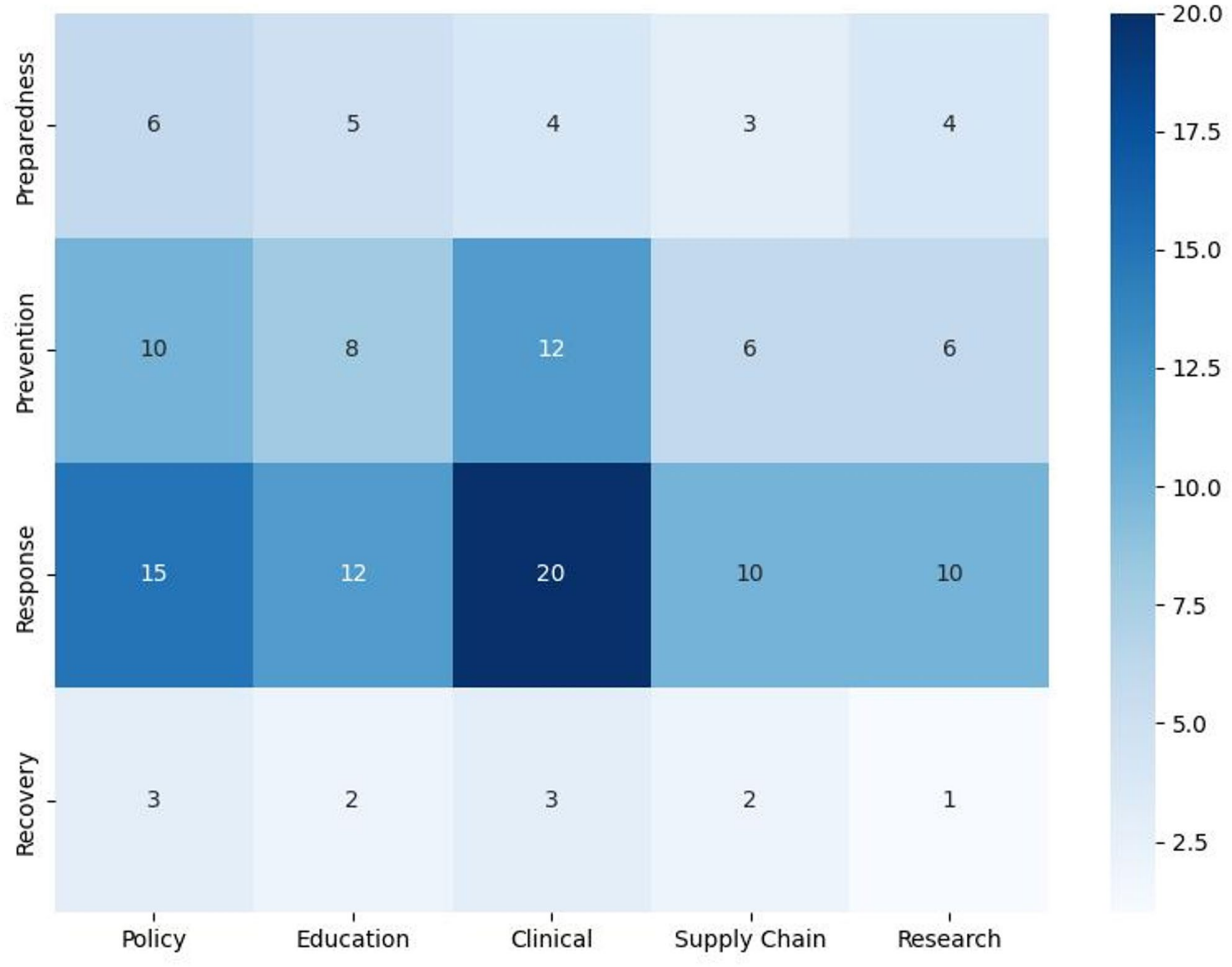
Heatmap of themes by stage

The roles were delivered within five main themes of activity and spread across the stages of the emergency cycle (Fig. 3).

### Sub-analysis

**Fig. 4:**
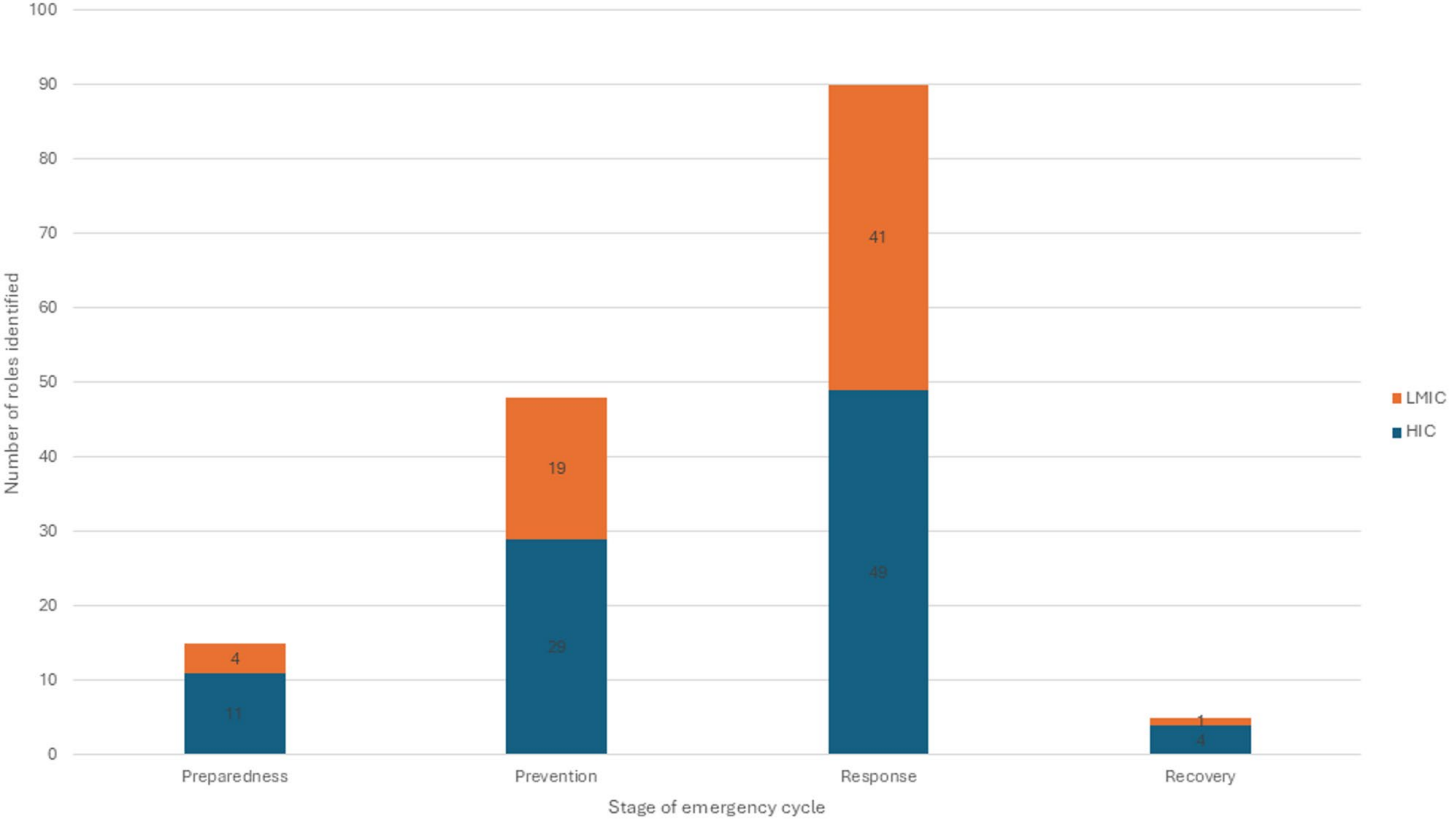
Sub-analysis of roles by country

When analysed by country, a broader range of roles were identified in high-income countries (HICs) compared to low- and middle-income countries (LMICs) (93 versus 65) (Fig. 4).

Due to the limited number of articles addressing outbreaks related to food or water contamination, bioterrorism, chemical or radioactive outbreaks, or poisonings (*n* = 10), sub-analysis by outbreak type was not feasible. Similarly, sub-analysis by pharmacy team member could not be performed because of insufficient data for non-pharmacists (*n* = 9).

### Secondary outcomes

#### Contribution related to health inequalities

Approximately a third of articles reported pharmacy contributions related to health inequalities (55/161, 34%) [21, 22, 28–33, 36, 40, 44, 46, 48, 55, 57, 59–62, 64, 69, 70, 81, 83, 85, 90–95, 99, 103, 107, 105, 111, 115, 131, 132, 134, 136, 137, 139–142, 144, 155, 157, 160, 161, 166, 167, 173, 177, 178].

Six key themes related to health inequalities were identified:

- Increasing accessibility to vaccinations – Support for vaccination efforts in rural, remote, and underserved areas, as well as among refugees, displaced individuals, and residents of long-term care facilities.
- Reducing inequity in healthcare provision – Contributions through frontline services in underserved settings, extended hours and improved accessibility.
- Novel solutions to reduce inequities – Strategies included virtual clinical trial systems, telephone clinics for follow-up, innovative medicine supply chains, and community pharmacy-based prevention and treatment.
- Supporting vulnerable patient groups – Interventions targeted homeless individuals, people who inject drugs (PWID), long-term care patients, and those facing gender disparities or stigma, including individuals with HIV and men who have sex with men.
- Targeted messaging to high-risk populations – Engagement in tailored communication for “unreached” groups, preventative advice, and collaboration with local communities for targeted messaging.
- Managing cultural differences and sensitivities – Activities included culturally appropriate messaging, addressing religious restrictions and beliefs, and working with community and faith groups.

#### Barriers to roles

Barriers to roles were discussed in nearly two thirds of the articles (100/161, 62.1%) [21, 22, 26, 28–30, 32, 34, 37, 39, 41, 42, 44–46, 48, 49, 51–58, 60–62, 64, 66, 67, 69–71, 75, 77, 78, 80, 81, 83, 87, 90–93, 95–98, 105, 108, 109, 112–117, 119–127, 129, 134, 135–138, 140, 142, 144–147, 149–160, 162–164, 166, 167, 173, 174, 176, 178]. These barriers were grouped within five key themes;

- Knowledge and training gaps included limited understanding of disease transmission, prevention, and vaccination, as well as insufficient formal training in emergency preparedness and public health roles.
- Regulatory and policy barriers included restrictive vaccination authority, unclear liability frameworks, and lack of formal agreements for pharmacy organisations or pharmacy professionals’ involvement in emergency planning.
- Resource and infrastructure constraints such as workforce shortages, supply chain disruptions, inadequate physical space, and financial limitations were frequently reported.
- Communication and coordination issues involved poor integration with public health systems, unclear roles, and underutilisation of pharmacy data for surveillance.
- Finally, cultural and social factors included public distrust, misinformation, stigma toward vulnerable populations, and inter-professional tensions.

## Discussion

This rapid systematic review based on empirical and non-empirical literature across all WHO regions identified 145 thematic derived role categories for pharmacists and their teams in non-COVID outbreaks. These were classified across five key themes - policy, education, supply chain, clinical, and research. The role categories spanned all stages of the WHO emergency cycle and all levels of intervention, however fewer roles were identified within the recovery stage, and at macro (system or national) level.

Most of the thematic roles identified were carried out within the response phase of the emergency cycle, aligning with international recommendations that prioritise pharmacy contributions to emergency response through supply chain management, triage support, and collaboration with public health teams [1]. The prevention stage emerged as the second most frequently reported area of pharmacy team involvement, reflecting roles in vaccination campaigns [20, 21, 28, 31, 33, 34, 36, 46, 48, 61, 64, 66, 67, 70, 72, 75, 83, 91–96, 103, 127, 138, 147, 155, 161, 177], antimicrobial stewardship [91, 131, 151], and public education aimed at reducing transmission [87, 147]. There were fewer roles identified in the preparedness and recovery stages. This could suggest a research gap: while there is evidence that pharmacy teams engage in preparedness activities [26, 37, 39, 42, 49, 52, 54, 74, 81, 85, 86, 88, 89, 96, 99, 100, 102, 105, 110–112, 119, 128, 131, 135, 136, 144, 148, 152, 154, 163, 176, 179] and post-outbreak rehabilitation [36, 41, 43, 51, 52, 62, 118, 128, 131, 132, 144, 148, 179] these efforts are underrepresented in published work, and this could indicate that the expertise of pharmacy professionals is being underutilised in these stages. This may limit opportunities for the full development of this professional identity and constrain recognition of pharmacy expertise beyond direct patient care. Addressing this gap is essential to fully capture the scope of pharmacy practice in outbreak resilience and to inform policy frameworks that integrate pharmacy professionals across all phases of emergency planning.

Most of the roles identified took place at the meso- (organisational/regional) level. This finding contrasts with previous literature which suggests that micro-level pharmaceutical public health roles such as direct patient care are typically more prevalent [4]. One possible explanation for this is that nearly a third of the included articles in this rapid systematic review did not specify the individual’s role but instead described the setting, such as the role of a hospital pharmacy within a hospital organisation responding to an outbreak (see Table 2). In such cases, outbreak‑related activities reflected pharmacy organisational functions or broader health‑system responses, rather than clearly delineated roles undertaken by individual pharmacists or pharmacy team members. At an organisational level, pharmaceutical services are often delivered by a pharmacy team that may include pharmacists, registered or regulated pharmacy technicians, assistants, trainees, students, and others. However, a lack of differentiation in the published literature may obscure the distinct contributions of different members of the pharmacy workforce within broader public health and emergency contexts. Improved differentiation may support clearer articulation of the specific contributions made by pharmacists and pharmacy team members, facilitate the development of professional identities of the pharmacy team members as public health practitioners, and enhance understanding of their respective roles within multidisciplinary teams as part of outbreak management. This is particularly important at a time when roles are evolving, including the expansion of responsibilities for pharmacy technicians in some countries [180–183]. Notably, this review identified only nine articles that focused exclusively on roles undertaken by pharmacy team members other than pharmacists [28, 49, 123, 143, 150, 152, 156, 163, 165]. These findings highlight a pressing need for further research and policy development to support recognition and advancement of all pharmacy team members, ensuring the full potential of the pharmacy workforce is leveraged in outbreak management.

Despite the high overall number of meso-level roles, there was a predominance towards high numbers of reported micro-level roles within the response and prevention stages, and within the clinical theme. This demonstrates the patient-focused nature of the work undertaken by most pharmacy professionals but reflects a narrow scope compared to the broader potential of pharmacy in public health. Previous work on pharmaceutical public health has demonstrated a need to expand pharmacy involvement beyond micro-level interventions to strengthen health system resilience [4]; this current review supports their recommendations to increase workforce capability. These recommendations include developing specialist public health career pathways, integrating pharmacists into multidisciplinary teams at all levels, and increasing access to public health training [4]. Intentional integration of outbreak‑related competencies within undergraduate and postgraduate education, experiential training, and career pathways may support professional confidence, interprofessional standing, and workforce preparedness. Embedding these roles as part of routine practice, rather than exceptional emergency responses, may also strengthen professional identity formation across the pharmacy workforce and better position pharmacy teams for future public health emergencies.

Macro-level roles, including policy development, advocacy, and participation in national outbreak planning, were less commonly documented. This imbalance may be due to limited publication of strategic and system-level activities or under-recognition of pharmacists’ contributions beyond direct patient care.

### By country income

Although studies from all World Health Organization regions were represented, the evidence base was unevenly distributed across geographical and income settings. A greater proportion of included articles originated from high‑income countries (HICs), with more limited representation from low/middle‑income settings (LMICs). However despite this limitation this rapid systematic review found that in LMICs, reported pharmaceutical public health roles tended to be concentrated at the micro-level, with a strong emphasis on patient-centred care, such as medicines and preventative treatment supply [96, 110–112, 122, 124, 154, 160, 164, 166, 170, 176, 178], patient counselling on treatments [76, 96, 120, 153, 158, 164, 173, 177] and basic infection prevention measures [114, 150, 155]. This reflects the accessibility of pharmacies as a first point of contact for many communities [184].

Lower numbers of reported meso- and macro- level roles in LMICs could be due to structural and regulatory constraints. In many settings, scope-of-practice regulations narrowly define pharmacists’ responsibilities, potentially restricting their involvement in surveillance, vaccination delivery, emergency preparedness planning, and health system coordination [185]. These regulatory limitations may be compounded by weak formal integration of pharmacists into national emergency governance structures, possibly limiting opportunities for participation in multidisciplinary decision-making and system-level response activities [8]. Along with other financial barriers such as pharmacy workforce shortages, uneven geographic distribution of professionals, and limited access to specialist public health or emergency preparedness training [186], pharmacy practice in outbreak management seems to be oriented towards immediate patient needs rather than population-level risk mitigation and system resilience, despite the heightened vulnerability of LMIC contexts to outbreaks and health emergencies [187]. Targeted capacity building in areas such as emergency preparedness, disease surveillance, risk communication, and supply chain management could enable pharmacy professionals to engage more effectively at meso and macro levels. Task shifting and task sharing strategies, supported by appropriate regulatory reform, may offer pragmatic mechanisms to expand public health functions within existing workforce constraints. Crucially, greater policy integration through the formal inclusion of pharmacists in national and subnational emergency preparedness and response frameworks could enhance coordination, improve continuity of essential medicines, and strengthen community-level resilience.

### Secondary outcomes

This review suggests that pharmacists and pharmacy teams have roles in non-COVID outbreaks that contribute to addressing health inequalities. Discussions of increased access to treatment, advice, and vaccinations highlights pharmacists and their teams as highly accessible healthcare providers [28, 31, 33, 48, 60, 64, 70, 81, 83, 93–95, 103, 109, 131, 132, 161, 173]. There are also reports of interventions targeted to high-risk, vulnerable, and hard-to-reach populations [21, 22, 40, 44, 46, 55, 57, 61, 62, 91, 136, 137, 155, 177]. The use of innovative solutions could mitigate the impact of outbreaks on the most disadvantaged groups and improves access to essential care [99, 141]. Contributions to inclusivity and cultural sensitivity emerged as key aspects, underscoring the importance of ensuring pharmacy professionals are well-informed, culturally competent, and equipped to reduce stigma while engaging marginalised communities effectively [144, 155, 157].

The barriers identified in this review, including regulatory constraints, training alignment, infrastructure gaps, and information-sharing and communication issues, are cross-cutting barriers that reflect broader health system design issues rather than profession-specific challenges. Addressing these barriers could have important implications for effective emergency preparedness, workforce planning, and equity-oriented public health policy well beyond pharmacy practice alone. Targeted health policy and management interventions could strengthen pharmacy contributions during public health emergencies; leveraging health systems to enable an expanded remit for pharmacy professionals [28, 37, 128, 148], improving integration of pharmacy into the emergency preparedness and public health planning at national and international levels [46, 51, 55], and ensuring sustainable funding mechanisms [87, 166] could improve public health in response to outbreaks and reduce inequalities. Multidisciplinary integration could strengthen system resilience, particularly in LMICs where workforce flexibility, task-shifting, and community reach are critical. In addition, embedding comprehensive education and training throughout the pharmacy career pathway is necessary to support development of pharmacy team knowledge and confidence in outbreaks [29, 39, 49, 57, 96, 124, 149, 152].

### Limitations

This rapid review prioritised timeliness over comprehensiveness. To ensure current relevance, empirical and non-empirical publications from 2014 onward were included; grey literature was excluded, along with articles that could not be retrieved (*n* = 32, see Additional Information Table S3). We also reduced independent verification at screening, extraction, and synthesis stages to 10%. A rapid review was deemed appropriate because providing a timely overview of pharmacy professionals’ roles in outbreaks to inform policy development was considered more important than achieving exhaustive coverage. Nonetheless, it remains possible that some roles or implications on barriers or health inequalities may have been missed or misinterpreted. However, the repeated description of similar roles and themes suggests that saturation may have been achieved.

The broad inclusion criteria relating to both publication type and the definition of pharmacy team members may have resulted in some duplication of reported roles, contributions to health inequalities, and barriers to delivery. However, the use of thematic coding to categorise findings, alongside narrative synthesis rather than frequency‑based reporting, was intentionally employed to mitigate these effects. Consequently, this is unlikely to have substantially influenced the overall conclusions.

The overall quality of the included articles were assessed as moderate based on the risk of bias assessment, partly reflecting the inclusion of non-empirical articles (46% of the included articles). However, this likely outcome had been considered in the methodology of the rapid review and, as the primary objective of this review was to identify the roles of pharmacy professionals in non-COVID outbreaks rather than to evaluate the impact of those roles or the outcomes reported, the methodological quality of individual studies was considered to pose a low risk to the validity of the conclusions.

Some non-empirical articles did not clearly distinguish between enacted outbreak activities and developmental or aspirational roles. Although roles explicitly described as unimplemented were excluded, some coding was based on authors’ descriptions of mixed practice and perspectives; however, because analysis was thematic and narrative rather than frequency‑based, this is unlikely to have materially affected the overall findings.

We also acknowledge limitations in the coding and classification approach. Some roles naturally span multiple stages of the emergency cycle, and although roles were classified based on descriptions in the original articles, overlap and fluidity between stages may have influenced categorisation. In addition, the inductive coding may not have fully captured authors’ original intentions, and reliance on translated texts for non-English articles may have limited the capture of contextual or linguistic nuance. This could potentially have affected interpretation of role categorisation or discussions of health inequalities and barriers. These limitations were partially mitigated through clinical expertise of the primary coder and second checking of 10% of the coding, however may not have been fully resolved.

The income‑based subgroup analysis also has limitations. Country classification was based on the 2025 World Bank income categories to ensure consistency and relevance to contemporary health systems, however may not fully reflect the economic or health system context at the time some studies were conducted. In addition, studies reporting pharmacy roles across multiple countries were excluded from this analysis to avoid misclassification, which reduced the number of studies available for comparison. Despite representation across all WHO regions, the geographical and income distribution of the included studies was uneven, which may influence the extent to which the findings reflect experiences across different health system and resource contexts.

### Research Implications

The findings from this rapid systematic review highlight important implications for future research on pharmacy roles in non-COVID outbreaks. In particular, research should address gaps in evidence relating to reporting of macro-level roles and those within the preparedness and recovery phases of the WHO emergency cycle. Addressing concerns about roles of various pharmacy team members and their professional identity within outbreak management and the wider public health context is also a priority. Although COVID-19 was outside the scope of this review, post-pandemic developments such as pharmacist prescribing of Paxlovid^®^ [188] and expanded pharmacy technician roles [180–183] illustrate how scopes of practice can evolve during and after public health emergencies, and future research should incorporate learning from COVID-19 alongside evidence from other outbreak events and this review. These will help to inform sustainable integration of pharmacy team members into routine outbreak preparedness, response, and recovery, including in LMIC settings and with explicit consideration of impacts on health inequalities.

### Policy Implications

The findings of this rapid systematic review suggest some key considerations for national and international policy makers, and pharmacy professional bodies. These also give additional support to existing recommendations from WHO.

At the national level, health authorities should consider how to further integrate pharmacy roles into emergency preparedness and response frameworks, supported by regulatory reform to expand scope of practice where needed to enable pharmacy teams to deliver vaccinations, manage emergency medicines, and participate in robust surveillance measures [8, 189, 190].

Internationally, collaboration between governments, global health organisations, and professional bodies is important to facilitate shared learning, aligning approaches, and supporting capacity development, particularly in low- and middle-income countries [191]. Embedding comprehensive education and training throughout pharmacy career pathways could ensure pharmacists are equipped for outbreak management and public health leadership [192]. Leveraging this expertise may contribute to enhanced preparedness, optimised patient outcomes, and mitigation of the disproportionate impact of future outbreaks on vulnerable populations worldwide.

Pharmacy professional bodies are well placed to support the integration of outbreak management and public health competencies into education and training pathways, to contribute to dialogue and advocate for policy changes that recognise advanced pharmacy roles in outbreak management and public health, and develop evidence-based guidance to support integration of pharmacy professionals across all stages of the emergency cycle [8]. In addition, professional bodies may play a role in supporting public and patient awareness of pharmacy services during outbreaks, ensuring communities understand how pharmacists can contribute to prevention, treatment, and recovery.

## Conclusion

Pharmacy team members have roles during non-COVID outbreaks across all stages of the WHO emergency cycle at all levels. However, their contributions are less well documented in recovery and at macro-level. Integrating pharmacy roles into emergency frameworks, supported by regulatory reform, funding, and training, may support health-system resilience, and reduce health inequalities. 

## Supplementary Information

Supplementary Material 1.

## Data Availability

All data generated or analysed during this rapid systematic review are included in this published article and its supplementary information files.

## Abbreviations

HIC: High income country
HIV: Human immunodeficiency virus
LMIC: Low or middle income country
MDT: Multi-disciplinary team
MMAT: Mixed method appraisal tool
PRISMA: Preferred Reporting Items for Systematic Reviews and Meta-Analyses
PWID: People who inject drugs
STD: Sexually Transmitted Disease
WHO: World Health Organization
