# Supplementary material for: The role of pharmacists and pharmacy team members in the prevention, preparedness, response and recovery of outbreaks – a global rapid systematic review
Source: BMC Public Health. 2026 Jun 5;26:2323. doi: 10.1186/s12889-026-27937-6 (PMC13459953; doi:10.1186/s12889-026-27937-6)
Supplement: Supplementary file 1 [file 12889_2026_27937_MOESM1_ESM.docx]

**Additional Information**

S1. Prisma checklist

| **Section and Topic** | **Item #** | **Checklist item** | **Location where item is reported** |
| --- | --- | --- | --- |
| **TITLE** | | |  |
| Title | 1 | Identify the report as a systematic review. | Page 1 – title |
| **ABSTRACT** | | |  |
| Abstract | 2 | See the PRISMA 2020 for Abstracts checklist. | Page 2 - Abstract |
| **INTRODUCTION** | | |  |
| Rationale | 3 | Describe the rationale for the review in the context of existing knowledge. | Page 4 and 5 - Introduction |
| Objectives | 4 | Provide an explicit statement of the objective(s) or question(s) the review addresses. | Page 5 -Introduction |
| **METHODS** | | |  |
| Eligibility criteria | 5 | Specify the inclusion and exclusion criteria for the review and how studies were grouped for the syntheses. | Methods - page 6 and 7. |
| Information sources | 6 | Specify all databases, registers, websites, organisations, reference lists and other sources searched or consulted to identify studies. Specify the date when each source was last searched or consulted. | Method – Page 6 |
| Search strategy | 7 | Present the full search strategies for all databases, registers and websites, including any filters and limits used. | Table 1 (Page 6) and Additional information |
| Selection process | 8 | Specify the methods used to decide whether a study met the inclusion criteria of the review, including how many reviewers screened each record and each report retrieved, whether they worked independently, and if applicable, details of automation tools used in the process. | Page 7 - Method |
| Data collection process | 9 | Specify the methods used to collect data from reports, including how many reviewers collected data from each report, whether they worked independently, any processes for obtaining or confirming data from study investigators, and if applicable, details of automation tools used in the process. | Page 7 and 8 -Method |
| Data items | 10a | List and define all outcomes for which data were sought. Specify whether all results that were compatible with each outcome domain in each study were sought (e.g. for all measures, time points, analyses), and if not, the methods used to decide which results to collect. | Page 8 - Method |
|  | 10b | List and define all other variables for which data were sought (e.g. participant and intervention characteristics, funding sources). Describe any assumptions made about any missing or unclear information. | n/a |
| Study risk of bias assessment | 11 | Specify the methods used to assess risk of bias in the included studies, including details of the tool(s) used, how many reviewers assessed each study and whether they worked independently, and if applicable, details of automation tools used in the process. | Page 8 - Method |
| Effect measures | 12 | Specify for each outcome the effect measure(s) (e.g. risk ratio, mean difference) used in the synthesis or presentation of results. | n/a |
| Synthesis methods | 13a | Describe the processes used to decide which studies were eligible for each synthesis (e.g. tabulating the study intervention characteristics and comparing against the planned groups for each synthesis (item #5)). | Page 8 and 9 – Method |
|  | 13b | Describe any methods required to prepare the data for presentation or synthesis, such as handling of missing summary statistics, or data conversions. | n/a |
|  | 13c | Describe any methods used to tabulate or visually display results of individual studies and syntheses. | Page 8 - Method |
|  | 13d | Describe any methods used to synthesize results and provide a rationale for the choice(s). If meta-analysis was performed, describe the model(s), method(s) to identify the presence and extent of statistical heterogeneity, and software package(s) used. | Page 8 - Method |
|  | 13e | Describe any methods used to explore possible causes of heterogeneity among study results (e.g. subgroup analysis, meta-regression). | n/a |
|  | 13f | Describe any sensitivity analyses conducted to assess robustness of the synthesized results. | n/a |
| Reporting bias assessment | 14 | Describe any methods used to assess risk of bias due to missing results in a synthesis (arising from reporting biases). | Page 8 – Method |
| Certainty assessment | 15 | Describe any methods used to assess certainty (or confidence) in the body of evidence for an outcome. | n/a |
| **RESULTS** | | |  |
| Study selection | 16a | Describe the results of the search and selection process, from the number of records identified in the search to the number of studies included in the review, ideally using a flow diagram. | Figure 1 (page 10) |
|  | 16b | Cite studies that might appear to meet the inclusion criteria, but which were excluded, and explain why they were excluded. | Figure 1 (page 10) and S3 |
| Study characteristics | 17 | Cite each included study and present its characteristics. | Table 1 (page 11) and Additional information |
| Risk of bias in studies | 18 | Present assessments of risk of bias for each included study. | Page 12 - Results and Additional information |
| Results of individual studies | 19 | For all outcomes, present, for each study: (a) summary statistics for each group (where appropriate) and (b) an effect estimate and its precision (e.g. confidence/credible interval), ideally using structured tables or plots. | Additional information |
| Results of syntheses | 20a | For each synthesis, briefly summarise the characteristics and risk of bias among contributing studies. | Table 1 (page 11) |
|  | 20b | Present results of all statistical syntheses conducted. If meta-analysis was done, present for each the summary estimate and its precision (e.g. confidence/credible interval) and measures of statistical heterogeneity. If comparing groups, describe the direction of the effect. | n/a |
|  | 20c | Present results of all investigations of possible causes of heterogeneity among study results. | n/a |
|  | 20d | Present results of all sensitivity analyses conducted to assess the robustness of the synthesized results. | n/a |
| Reporting biases | 21 | Present assessments of risk of bias due to missing results (arising from reporting biases) for each synthesis assessed. | Page 12 and additional information |
| Certainty of evidence | 22 | Present assessments of certainty (or confidence) in the body of evidence for each outcome assessed. | Page 12 and additional information |
| **DISCUSSION** | | |  |
| Discussion | 23a | Provide a general interpretation of the results in the context of other evidence. | Page 18 and 19 - Discussion |
|  | 23b | Discuss any limitations of the evidence included in the review. | Page 23 - Discussion |
|  | 23c | Discuss any limitations of the review processes used. | Page 23 - Discussion |
|  | 23d | Discuss implications of the results for practice, policy, and future research. | Page 24 - Discussion |
| **OTHER INFORMATION** | | |  |
| Registration and protocol | 24a | Provide registration information for the review, including register name and registration number, or state that the review was not registered. | Page 6 - Method |
|  | 24b | Indicate where the review protocol can be accessed, or state that a protocol was not prepared. | n/a |
|  | 24c | Describe and explain any amendments to information provided at registration or in the protocol. | Page 5 - Method |
| Support | 25 | Describe sources of financial or non-financial support for the review, and the role of the funders or sponsors in the review. | Page 26 - Funding |
| Competing interests | 26 | Declare any competing interests of review authors. | Page 26 - Competing interests |
| Availability of data, code and other materials | 27 | Report which of the following are publicly available and where they can be found: template data collection forms; data extracted from included studies; data used for all analyses; analytic code; any other materials used in the review. | n/a |

S2. Searches

Embase

| 1 | exp pharmacist/ | 107014 |
| --- | --- | --- |
| 2 | pharmacist.ti,kw. | 18564 |
| 3 | exp pharmacy technician/ | 3158 |
| 4 | pharmacy technician.ti,kw. | 511 |
| 5 | exp pharmacy student/ | 10505 |
| 6 | pharmacy student.ti,kw. | 717 |
| 7 | pharmacy staff.kw,ti. | 306 |
| 8 | exp "pharmacy (shop)"/ | 33200 |
| 9 | exp pharmacy school/ | 1586 |
| 10 | exp clinical pharmacy/ | 12548 |
| 11 | exp pharmaceutical care/ | 25327 |
| 12 | exp hospital pharmacy/ | 16124 |
| 13 | exp pharmacy research/ | 299 |
| 14 | exp pharmacy practice/ | 2423 |
| 15 | exp pharmacist attitude/ | 4954 |
| 16 | exp pharmacist patient relationship/ | 216 |
| 17 | exp epidemic/ | 139122 |
| 18 | exp pandemic/ | 214809 |
| 19 | exp pandemic preparedness/ | 342 |
| 20 | outbreak*.ti,kw. | 49898 |
| 21 | pandemic*.ti,kw. | 124358 |
| 22 | epidemic*.ti,kw. | 41295 |
| 23 | exp biological accident/ | 379 |
| 24 | exp chemical accident/ | 765 |
| 25 | exp disaster management/ | 20132 |
| 26 | 17 or 18 or 19 or 20 or 21 or 22 or 23 or 24 | 396653 |
| 27 | exp anthrax/ | 6995 |
| 28 | exp Sverdlovsk anthrax outbreak/ | 12 |
| 29 | exp influenza/ | 116316 |
| 30 | exp chikungunya/ | 6701 |
| 31 | exp cholera/ | 11756 |
| 32 | exp Crimean Congo hemorrhagic fever/ | 1864 |
| 33 | exp dengue/ | 31663 |
| 34 | exp diphtheria/ | 10429 |
| 35 | exp Ebola hemorrhagic fever/ | 8600 |
| 36 | exp hepatitis/ | 384593 |
| 37 | exp measles/ | 24217 |
| 38 | exp meningitis/ | 115590 |
| 39 | exp Middle East respiratory syndrome coronavirus/ | 5956 |
| 40 | exp plague/ | 7212 |
| 41 | exp poliomyelitis/ | 17440 |
| 42 | exp Rift Valley fever/ | 1527 |
| 43 | exp severe acute respiratory syndrome/ | 11856 |
| 44 | exp typhoid fever/ | 12939 |
| 45 | exp hemorrhagic fever/ | 21372 |
| 46 | exp yellow fever/ | 5583 |
| 47 | exp Zika virus/ | 10835 |
| 48 | pharmacy.ti,kw. | 43291 |
| 49 | 1 or 2 or 3 or 4 or 5 or 6 or 7 or 8 or 9 or 10 or 11 or 12 or 13 or 14 or 15 or 16 or 48 | 178139 |
| 50 | exp Human immunodeficiency virus/ | 228363 |
| 51 | exp sexually transmitted disease/ | 117600 |
| 52 | 27 or 28 or 29 or 30 or 31 or 32 or 33 or 34 or 35 or 36 or 37 or 38 or 39 or 40 or 41 or 42 or 43 or 44 or 45 or 46 or 47 or 50 or 51 | 1045911 |
| 53 | 26 or 52 | 1370430 |
| 54 | covid.m_titl. | 329104 |
| 55 | exp radiation accident/ | 2266 |
| 56 | 53 or 55 | 1372668 |
| 57 | 49 and 56 | 8830 |
| 58 | 57 not 54 | 6657 |
| 59 | limit 58 to (english language and yr="2014 -Current") | 4719 |
| 60 | 26 or 55 | 398900 |
| 61 | 49 and 60 | 4292 |
| 62 | 61 not 54 | 2191 |
| 63 | limit 62 to (english language and yr="2014 -Current") | 1799 |
| 64 | increased incidence.mp. | 51655 |
| 65 | increased prevalence.mp. | 27494 |
| 66 | 26 or 55 or 64 or 65 | 476013 |
| 67 | 49 and 66 | 4448 |
| 68 | "covid*".m_titl. | 331436 |
| 69 | "corona*".m_titl. | 372558 |
| 70 | 54 or 68 or 69 | 695152 |
| 71 | 67 not 70 | 2219 |
| 72 | limit 71 to yr="2014 -Current" | 1864 |
| 73 | 52 or 66 | 1446474 |
| 74 | 49 and 73 | 8981 |
| 75 | 74 not 70 | 6676 |
| 76 | limit 75 to yr="2014 -Current" | 4844 |
| 77 | exp monkeypox/ | 5019 |
| 78 | 52 or 77 | 1049711 |
| 79 | 17 or 19 or 20 or 22 or 23 or 24 | 178301 |
| 80 | 78 or 79 | 1174569 |
| 81 | 49 and 80 | 5630 |
| 82 | 81 not 70 | 5299 |
| 83 | limit 82 to yr="2014 -Current" | 3537 |
| 84 | 17 or 19 or 20 or 22 or 23 or 24 or 55 | 180642 |
| 85 | 49 and 84 | 871 |
| 86 | opioid.m_titl. | 63169 |
| 87 | 68 or 69 or 86 | 758440 |
| 88 | 85 not 87 | 592 |
| 89 | limit 88 to yr="2014 -Current" | 366 |
| 90 | obesity.m_titl. | 153532 |
| 91 | 87 or 90 | 909401 |
| 92 | 85 not 91 | 583 |
| 93 | limit 92 to yr="2014 -Current" | 359 |
| 94 | (increas* and (prevalen* or inciden*)).mp. [mp=title, abstract, heading word, drug trade name, original title, device manufacturer, drug manufacturer, device trade name, keyword heading word, floating subheading word, candidate term word] | 1242478 |
| 95 | (HIV or (human and immunodeficiency) or STD or (sexually and transmitted and (infection* or disease*)) or anthrax or Chikungunya or Cholera or "Crimean-Congo" or Dengue or Diphtheria or Ebola or Hepatitis or Influenza or Measles or Meningitis or (Middle and East and respiratory) or Plague or Poliomyelitis or Rift valley fever or (Severe and acute and respiratory) or Typhoid or (Yellow and fever) or Zika or mpox or "m-pox" or monkeypox or (resistan* and infection)).mp. [mp=title, abstract, heading word, drug trade name, original title, device manufacturer, drug manufacturer, device trade name, keyword heading word, floating subheading word, candidate term word] | 2086825 |
| 96 | ((increas* and (prevalen* or inciden*)) adj5 (HIV or (human and immunodeficiency) or STD or (sexually and transmitted and (infection* or disease*)) or anthrax or Chikungunya or Cholera or "Crimean-Congo" or Dengue or Diphtheria or Ebola or Hepatitis or Influenza or Measles or Meningitis or (Middle and East and respiratory) or Plague or Poliomyelitis or Rift valley fever or (Severe and acute and respiratory) or Typhoid or (Yellow and fever) or Zika or mpox or "m-pox" or monkeypox or (resistan* and infection))).tw,kf. | 55926 |
| 97 | 84 or 96 | 233010 |
| 98 | 49 and 97 | 1102 |
| 99 | 98 not 91 | 804 |
| 100 | limit 99 to yr="2014 -Current" | 524 |

Medline

| 1 | exp Pharmacists/ | 23452 |
| --- | --- | --- |
| 2 | pharmacist*.ti,kw. | 21317 |
| 3 | exp Pharmacy Technicians/ | 940 |
| 4 | pharmacy technician.ti,kw. | 273 |
| 5 | exp Students, Pharmacy/ | 4943 |
| 6 | pharmacy student.ti,kw. | 476 |
| 7 | pharmacy staff.ti,kw. | 156 |
| 8 | exp Pharmacy/ | 10744 |
| 9 | exp Community Pharmacy Services/ | 6195 |
| 10 | exp Pharmacy Service, Hospital/ | 12913 |
| 11 | exp Pharmacies/ | 10170 |
| 12 | exp Pharmaceutical Services, Online/ | 138 |
| 13 | exp Pharmacy Residencies/ | 445 |
| 14 | exp Pharmaceutical Services/ | 84748 |
| 15 | exp Schools, Pharmacy/ | 1704 |
| 16 | exp Pharmacy Research/ | 140 |
| 17 | pharmacy.kw,ti. | 25250 |
| 18 | 1 or 2 or 3 or 4 or 5 or 6 or 7 or 8 or 9 or 10 or 11 or 12 or 13 or 14 or 15 or 16 or 17 | 122969 |
| 19 | exp Disease Outbreaks/ | 243394 |
| 20 | exp Epidemics/ | 152147 |
| 21 | exp Pandemics/ | 136917 |
| 22 | epidemic*.ti,kw. | 47002 |
| 23 | pandemic*.ti,kw. | 113565 |
| 24 | outbreak*.ti,kw. | 48206 |
| 25 | exp Pandemic Preparedness/ | 10915 |
| 26 | exp Disasters/ | 116605 |
| 27 | exp Chemical Hazard Release/ | 628 |
| 28 | exp Biohazard Release/ | 156 |
| 29 | exp Radioactive Hazard Release/ | 8459 |
| 30 | 19 or 20 or 21 or 22 or 23 or 24 or 25 or 27 or 28 or 29 | 343999 |
| 31 | exp HIV/ | 110088 |
| 32 | exp Sexually Transmitted Diseases/ | 424254 |
| 33 | exp Anthrax/ | 4634 |
| 34 | exp Influenza, Human/ | 60776 |
| 35 | exp Chikungunya Fever/ | 3181 |
| 36 | exp Cholera/ | 9580 |
| 37 | exp Hemorrhagic Fever, Crimean/ | 1386 |
| 38 | exp Dengue/ | 17295 |
| 39 | exp Diphtheria Toxin/ | 3691 |
| 40 | exp Hemorrhagic Fever, Ebola/ | 6951 |
| 41 | exp Hepatitis/ | 188244 |
| 42 | exp Measles/ | 17175 |
| 43 | exp Meningitis/ | 59904 |
| 44 | exp Middle East Respiratory Syndrome Coronavirus/ | 2173 |
| 45 | exp Plague/ | 5683 |
| 46 | exp Poliomyelitis/ | 20909 |
| 47 | exp Rift Valley Fever/ | 1425 |
| 48 | exp Severe Acute Respiratory Syndrome/ | 5762 |
| 49 | exp Typhoid Fever/ | 11856 |
| 50 | exp Hemorrhagic Fevers, Viral/ | 35768 |
| 51 | exp Yellow Fever/ | 3567 |
| 52 | exp Zika Virus/ | 7256 |
| 53 | 31 or 32 or 33 or 34 or 35 or 36 or 37 or 38 or 39 or 40 or 41 or 42 or 43 or 44 or 45 or 46 or 47 or 48 or 49 or 50 or 51 or 52 | 865428 |
| 54 | 30 or 53 | 1151043 |
| 55 | 18 and 54 | 4346 |
| 56 | covid.m_titl. | 289501 |
| 57 | 55 not 56 | 3446 |
| 58 | limit 57 to (english language and yr="2014 -Current") | 2011 |
| 59 | 18 and 30 | 1794 |
| 60 | 59 not 56 | 905 |
| 61 | limit 60 to (english language and yr="2014 -Current") | 637 |
| 62 | increased incidence.mp. | 34746 |
| 63 | increased prevalence.mp. | 18347 |
| 64 | "covid*".m_titl. | 290610 |
| 65 | "corona*".m_titl. | 286390 |
| 66 | 56 or 64 or 65 | 569319 |
| 67 | 30 or 62 or 63 | 395800 |
| 68 | 18 and 67 | 1915 |
| 69 | 68 not 66 | 982 |
| 70 | limit 69 to yr="2014 -Current" | 666 |
| 71 | 53 or 67 | 1200750 |
| 72 | 18 and 71 | 4462 |
| 73 | 72 not 66 | 3515 |
| 74 | limit 73 to yr="2014 -Current" | 2081 |
| 75 | exp "Mpox (monkeypox)"/ | 3377 |
| 76 | 53 or 75 | 868373 |
| 77 | 19 or 20 or 22 or 24 or 25 or 27 or 28 or 29 | 297377 |
| 78 | 76 or 77 | 1108283 |
| 79 | 18 and 78 | 4039 |
| 80 | 79 not 66 | 3372 |
| 81 | limit 80 to yr="2014 -Current" | 2004 |
| 82 | 19 or 20 or 22 or 24 or 25 or 27 or 28 or 29 | 297586 |
| 83 | ((increas* and (prevalen* or inciden*)) adj5 (HIV or (human and immunodeficiency) or STD or (sexually and transmitted and (infection* or disease*)) or anthrax or Chikungunya or Cholera or "Crimean-Congo" or Dengue or Diphtheria or Ebola or Hepatitis or Influenza or Measles or Meningitis or (Middle and East and respiratory) or Plague or Poliomyelitis or Rift valley fever or (Severe and acute and respiratory) or Typhoid or (Yellow and fever) or Zika or mpox or "m-pox" or monkeypox or (resistan* and infection))).tw,kf. | 37994 |
| 84 | 82 or 83 | 332894 |
| 85 | obesity.m_titl. | 114390 |
| 86 | opioid.m_titl. | 48940 |
| 87 | 56 or 64 or 65 or 85 or 86 | 730826 |
| 88 | 18 and 84 | 1624 |
| 89 | 88 not 87 | 767 |
| 90 | limit 89 to yr="2014 -Current" | 471 |

SCOPUS

| ( ( ( TITLE-ABS-KEY ( pharmacist* ) ) OR ( TITLE-ABS-KEY ( pharmacy AND technician ) ) OR ( TITLE-ABS-KEY ( pharmacy AND student ) ) OR ( TITLE-ABS-KEY ( pharmacy AND staff ) ) OR ( TITLE-ABS-KEY ( pharmacy ) ) OR ( TITLE-ABS-KEY ( pharmacies ) ) ) AND ( ( ( TITLE-ABS-KEY ( outbreak* ) ) OR ( TITLE-ABS-KEY ( epidemic* ) ) ) OR ( ( TITLE-ABS-KEY ( "increas* incidenc*" W/5 hiv OR "human immunodeficiency" OR std OR "sexually transmitted" OR anthrax OR chikungunya OR cholera OR "crimean-congo" OR dengue OR diphtheria OR ebola OR hepatitis OR influenza OR measles OR meningitis OR "middle east respiratory" OR plague OR poliomyelitis OR "rift valley fever" OR "severe acute respiratory" OR typhoid OR "yellow fever" OR zika OR mpox OR "m-pox" OR monkeypox OR resistan* ) ) OR ( TITLE-ABS-KEY ( "increas* prevalenc*" W/5 hiv OR "human immunodeficiency" OR std OR "sexually transmitted" OR anthrax OR chikungunya OR cholera OR "crimean-congo" OR dengue OR diphtheria OR ebola OR hepatitis OR influenza OR measles OR meningitis OR "middle east respiratory" OR plague OR poliomyelitis OR "rift valley fever" OR "severe acute respiratory" OR typhoid OR "yellow fever" OR zika OR mpox OR "m-pox" OR monkeypox OR resistan* ) ) ) ) ) AND NOT ( TITLE ( covid OR obesity OR opioid OR coronavirus OR tobacco ) ) AND PUBYEAR > 2013 AND PUBYEAR < 2026 | 829 |
| --- | --- |

S3. Articles that could not be retrieved.

| Author(s) | Article title | Year | Journal | Volume | Issue | Page |
| --- | --- | --- | --- | --- | --- | --- |
| Allen, Loyd V Jr | PreScription: Endemics, Epidemics, and Pandemics. | 2021 | International journal of pharmaceutical compounding | 25 | 4 | 268 |
| Bacci, Jennifer L and Odegard, Peggy and Arnold, Jenny and Stergachis, Andy | Strengthening pandemic preparedness through pharmacy and public health collaborations: Findings from a facilitated discussion exercise. | 2021 | Journal of the American Pharmacists Association : JAPhA | 61 | 3 | e99-e106 |
| Becskehazi-Tar A. and Kovacs Zs. and Molnar B. and Richter K. and Szabo A. | Challenges for the next quarter century. Quality in hospital pharmacy - hospital pharmaceutical quality development framework | 2020 | Acta Pharmaceutica Hungarica | 90 | 2 | 45 EP - 46 |
| Cabaleiro, J. | New England Compounding Center Indictment | 2015 | International journal of pharmaceutical compounding | 19 | 2 | 94-102 |
| Chaple, E.B. | Background and the beginnings of the institutionalization of hygiene and epidemiology in Cuba | 2020 | Anales de la Academia de Ciencias de Cuba | 10 | 1 |  |
| Chopra, D.S. and Kaul, A. | Strategies to overcome superbug invasions: Emerging research and opportunities | 2021 | Strategies to Overcome Superbug Invasions: Emerging Research and Opportunities |  |  | 1-319 |
| Dickson, W. and Meyer, J. and Leston, J. and Reilley, B. | Borne of necessity: pharmacy-based harm reduction and express sexually transmitted infection services | 2024 | Journal of the American Pharmacists Association | 64 | 6 |  |
| Faure S. and Apaire-Marchais V. and Buxeraud J. | Involvement of pharmacists in influenza immunisation | 2017 | Actualites Pharmaceutiques | 56 | 568 | 43 EP - 46 |
| Ford H. and Von Waldner T. and Perri M. | Pharmacists' roles in post-September 11th disasters: A content analysis of pharmacy literature | 2014 | Journal of Pharmacy Practice | 27 | 4 | 350 EP - 357 |
| Friedrich C. | Where the malady does not want to go away and very great need prevails: The service of pharmacists during the cholera epidemic in the 19th century | 2020 | Deutsche Apotheker Zeitung | 60 | 18 | 74 |
| Gomez, A.B. | The pen falls from the hand: recovery of the Memory of Malaga epidemics by the pharmacist Joaqua­n Garci­a Briz y Galindo | 2022 | Documenta et Instrumenta | 20 |  | 53-72 |
| Gaidarov, G.M. and Alekseevskaya, T.I. and Demidova, T.V. and Sofronov, O.Yu. | The state policy of the Russian Empire in the fight against the syphilis epidemic among foreign communities in the northeastern districts of Eastern Siberia in the 19th century | 2023 | Kazan Medical Journal | 104 | 5 | 778-789 |
| Glassgold, J.M. | Compounded drugs | 2014 | Drug Compounding: Background, Issues and FDA Oversight |  |  | 14977 |
| Goetz R. and Casavant M.J. and Spiller H.A. and Behrman A. | Survey of us poison center surge capacity planning | 2014 | Clinical Toxicology | 52 | 7 | 779 EP - 780 |
| Harale S. and Hupare P. and Ghatage P. and Govande V. and Gurav O. and Bandgar S. and Patil S. and Gaikwad D. | CHALLENGES AND OPPORTUNITIES WITH DRUG REPURPOSING: AN EMERGING TECHNIQUE IN DRUGS DISCOVERY | 2023 | Indian Drugs | 60 | 11 | 7 EP - 18 |
| He, L. and Zhang, K. and Zhang, W.-Q. and Wang, S.-N. and Zhang, W. and Zhang, Y.-Q. | Online Teaching Practice and Effect Evaluation of Biopharmaceutical Experiment Course during Epidemic Prevention | 2021 | Chinese Journal of Pharmaceutical Biotechnology | 28 | 4 | 411-413 |
| Hecht H. and Hayney M.S. | Understanding the role of vaccination exemption in the recent measles outbreaks | 2019 | Journal of the American Pharmacists Association | 59 | 5 | 753 EP - 755 |
| Jairoun, Ammar Abdulrahman | Responding to the World Health Organization's warning of a future pandemic: a call to preparatory action in pharmacy practice. | 2023 | The International journal of pharmacy practice | 31 | 6 | 567-568 |
| Jairoun, Ammar Abdulrahman and Al-Hemyari, Sabaa Saleh and Shahwan, Moyad and Zyoud, Sa'ed H and Abu-Gharbieh, Eman and Jairoun, Maimona and Kurdi, Amanj and Godman, Brian | Airport-based pharmacies: Products, services and challenges essential for patients in a global travel landscape. | 2023 | Journal of the American Pharmacists Association : JAPhA | 63 | 5 | 1500-1503 |
| Jamieson N. and Netongo P.M. and Dias A.G. and Ogunfowokan G. and Baker B. and Horby P. and Wilder-Smith A. and Lang T. | Regional networks for research capacity and why these are needed to be prepared for disease outbreaks | 2019 | Transactions of the Royal Society of Tropical Medicine and Hygiene | 113 |  | S5 |
| Jenkins, C. | Curing Venice's plagues: Pharmacology and witchcraft | 2017 | Postmedieval | 8 | 2 | 202-208 |
| Jia Y.-T. and Cai Y. and Liang B.-B. and Wang J. | Guidance for central pharmacy management of clinical drug research institutions based on grade I response to major public health emergencies | 2020 | Chinese Journal of New Drugs | 29 | 7 | 762 EP - 765 |
| Jin, L. and Yang, Y. and Tang, Y. and Huan, H. and Shen, Z. | Construction of quality standards for pharmacy management in public health emergencies | 2024 | Chinese Journal of General Practitioners | 23 | 8 | 881-885 |
| Kerkering, T.M. | An Iatrogenic Outbreak of Central Nervous System Fungal Infections Due To Contaminated Steroids: a Review of the Framework of Its Control and Management. What Did We Learn? | 2017 | Current Clinical Microbiology Reports | 4 | 2 | 71-77 |
| Kitzke T.N. and Hayney M.S. | Hepatitis A outbreaks | 2018 | Journal of the American Pharmacists Association | 58 | 3 | 319 EP - 320 |
| Komandzhaev, A.N. and Komandzhaev, E.A. and Miymanbaeva, F.N. | Public Healthcare System in Early 20th Century Kalmyk Steppe | 2021 | Oriental Studies | 14 | 5 | 919-928 |
| Mathews E. and James T. and Neill K. | Someone you love: Raising human papilloma-virus awareness | 2016 | Journal of the American Pharmacists Association | 56 | 3 | e119 EP - e120 |
| Schersch S. | End of the epidemic emergency situation: Special rules in pharmacies remain | 2021 | Pharmazeutische Zeitung | 166 | 43 | 6 |
| Tan, X.X. and Ye, M.-Y. and Un, Y.-Y. and Yang, Y.-W. and Kang, M. | Correlation analysis between ratio of over-the-counter pharmaceutical sales and influenza-like-illness cases | 2020 | Modern Preventive Medicine | 47 | 10 | 1733-1737 |
| Teshome B.F. and Reveles K.R. and Lee G.C. and Ryan L. and Frei C.R. | How gaps in regulation of compounding pharmacy set the stage for a multistate fungal meningitis outbreak | 2014 | Journal of the American Pharmacists Association | 54 | 4 | 441 EP - 445 |
| Vo, T.Q. | Knowledge, attitudes and practices related to dengue fever: The case of university pharmacy students in the highlands of Vietnam | 2018 | Eurasian Journal of Analytical Chemistry | 13 | 3 | 252 |
| Wong, M. and McPherson, T.D. and Lash, M. and Garcia, E. and Silverstein, M. and Alexander, M. and Thomas, G.P. and Foote, M.M.K. | New York City's Rapid Response to the 2022 Mpox Outbreak | 2024 | NEJM Catalyst Innovations in Care Delivery | 5 | 8 |  |

S4. Characteristics of studies

| Author(s) | Year | Reference | Type of paper | Country | HIC or LMIC | Setting | Pharmacy professional | Outbreak type | Roles identified | Discussion of issues relating to health inequalities | Barriers to delivery |
| --- | --- | --- | --- | --- | --- | --- | --- | --- | --- | --- | --- |
| Abbas, K.M. et al. | 2016 | 19 | Systematic review (JCI) | United States of America | HIC | Multiple | Not specified | Contamination of pharmaceutical product | Follow aseptic policy or guidance when making up pharmaceutical products - organisation Pharmaceutical product source investigation Recall of contaminated pharmaceutical products |  |  |
| Abu Esba, L.C. et al. | 2022 | 144 | Narrative (JCI) | Saudi Arabia | HIC | Hospital | Not specified | Poisoning | Patient education and advice - Symptom management Supply of treatment to patients (OTC, sales or dispensing) Stock management and distribution of treatments, preventative measures or consumables - organisation Post learning analysis and sharing - organisation | Ensuring antidote stocking and planning is tailored to address cultural differences, e.g. availability of ethanol. | Areas of weakness in stocking and access that were important to be reassessed and modified to be prepared for any further poisoning cases presenting. |
| Ade-adekunle, O.A. et al. | 2024 | 161 | Expert opinion (JCI) | Nigeria | LMIC | Retail/community | Not specified | Diphtheria | Vaccination First point of patient contact Public education - vaccination | Increased accessibility to vaccination in rural communities |  |
| Agaku, I. et al. | 2024 | 143 | Other | Multiple | n/a | Higher Education Institutions | Pharmacy faculty staff | Not specific - pandemic, epidemic etc | Provide education for pharmacy professionals in organisation |  |  |
| Ahmed, A. et al. | 2018 | 150 | Quantitative descriptive (MMAT) | Pakistan | LMIC | Higher Education Institutions | Pharmacy student | Crimean Congo Haemorrhagic Fever | Knowledge of IPC policy, guidance or recommendations Undertake Self education - about disease Knowledge about the disease/outbreak |  | The findings of this study indicate that the student healthcare workers knowledge about the infection was not optimal; however, their attitudes were positive. |
| Ahmed, M.A.E.-G.E.-S. et al. | 2024 | 126 | Narrative (JCI) | Multiple | n/a | Compounding pharmacy or centre | Not specified | Contamination of pharmaceutical product | Follow aseptic policy or guidance when making up pharmaceutical products - organisation |  | Poor handling, repackaging, and nonadherence to good manufacturing practices during dispensing and packaging have resulted in the microbiological contamination of non-sterile pharmaceutical products. |
| Ahmed, S.K. et al. | 2023 | 155 | Quantitative descriptive (MMAT) | Iraq | LMIC | Not specified | Pharmacist | Mpox or Monkeypox | Vaccination Follow IPC recommendations Knowledge about the disease/outbreak | Management of sensitive issues that are impacted by religious restrictions e.g. males participating in sexual activities with other men. Increased accessibility to vaccination and preventative advice for high-risk populations | Gaps in monkeypox knowledge were most conspicuous for issues of transmission from one person to another, how to differentiate between the clinical features of monkeypox and smallpox, specific vaccines, and treatment for monkeypox. They also need to have a good attitude to the disease - healthcare workers may use their credibility to promote vaccination through events, campaigns, and online forums. Furthermore, they can work together with community leaders, religious figures, and officials to advocate for vaccination, stressing the significance of stopping the spread of monkeypox. |
| Akiyode, O. et al. | 2019 | 20 | Expert opinion (JCI) | United States of America | HIC | Not specified | Pharmacist | Mumps | Vaccination Public education - vaccination Patient education and advice - symptoms |  |  |
| Al Awaidy, S. et al. | 2018 | 127 | Qualitative study (MMAT) | Multiple | n/a | Retail/community | Not specified | Influenza | Vaccination Vaccine dispensing |  | There is a need for determining the vaccine prescribers and vaccinators to estimate targets for awareness training as needed. |
| Aldohyan, M. et al. | 2019 | 145 | Quantitative descriptive (MMAT) | Saudi Arabia | HIC | Hospital | Pharmacist | Middle East Respiratory Syndrome (MERS) | Undertake self education - about disease Knowledge about the disease/outbreak |  | The level of knowledge on MERS-CoV among HCWs in primary healthcare clinics in this setting was found to be less than optimal. |
| Alhuzaimi A. et al. | 2024 | 146 | Quantitative descriptive (MMAT) | Saudi Arabia | HIC | Not specified | Pharmacist | Food or water contamination | Knowledge about the disease/outbreak |  | The overall HCWs’ botulism knowledge score was moderate highlighting that while foundational knowledge exists, there are significant gaps, especially regarding potential food sources, incubation period and presenting symptoms. |
| Alkhalili, M. et al. | 2017 | 128 | Mixed methods (MMAT) | Multiple | n/a | Not specified | Multiple | Not specific - pandemic, epidemic etc | Undertake self education - outbreak management Knowledge of managing outbreaks Development of organisational emergency planning policies and guidance Development of organisational treatment policy or guidance Provide education for pharmacy professionals in organisation - outbreak management Involvement in emergency planning at organisational level Development of legislation to support national emergency planning Get recommended vaccinations Maintain professional competence Take part in organisational preparedness simulations Business continuity planning to allow continuation of pharmacy services Consider the use of novel solutions to provide usual pharmacy services Follow IPC recommendations Follow IPC policy, guidance or recommendations Development of IPC policy or guidance - organisational Development of national emergency plans Pharmaceutical care of patients Patient education and advice - disease Prevent hoarding and diversion of medicines Develop, review or update legislation Maintain organisational compliance - with legislation Stay within law for supply of medicines Maintain accurate and complete patient records. Patient education and advice Communication - within organisation Communication at national level Pharmaceutical care of patients Supply of treatment to patients (OTC, sales or dispensing) Vaccine dispensing Vaccination Collaboration - with wider teams involved in response Collaboration at national level Personal composure Undertake expanded job roles Team spirit Maintain organisational compliance - with local guidance Stock management of medicines and consumables - organisation Continued supply of medicines for long-term conditions Undertake Self education - disease Pharmaceutical care of patients Resumption of usual activity Liaise with wider stakeholders regarding recovery Self-reflection Contribution to post learning analysis Post learning analysis and sharing - organisation Long-term follow up and management of lasting effects of outbreak Relocate personnel and equipment Review and update organisational protocol, policies, and guidelines Review and update organisational protocol, policies, and guidelines Shut down temporary premises Review legislation |  |  |
| Alkhorem, I.M. et al. | 2024 | 129 | Systematic review (JCI) | Multiple | n/a | Hospital | Pharmacist | Not specific - infectious diseases | Part of Infection, Prevention, and Control Multi-disciplinary Team Part of outbreak response Multi-Disciplinary Team |  | Resource limitations frequently emerged as a barrier in the studies reviewed. |
| Alrasheedy A.A. et al. | 2023 | 147 | Quantitative descriptive (MMAT) | Saudi Arabia | HIC | Retail/community | Pharmacist | Mpox or Monkeypox | Vaccination Patient education and advice - disease Public education - prevention and vaccination Knowledge about the disease/outbreak Vaccination - recognition of at risk patients to prioritise for vaccination |  | There is a need to improve community pharmacists’ knowledge in terms of disease management, prevention, vaccination, and modes of transmission. |
| Alsanafi, M. et al. | 2022 | 157 | Quantitative descriptive (MMAT) | Kuwait | HIC | Not specified | Pharmacist | Mpox or Monkeypox | Knowledge about the disease/outbreak Symptom recognition and diagnosis Knowledge about the disease/outbreak Knowledge of IPC policy, guidance or recommendations | Ensuring that high-risk patient populations or those with the disease are not stigmatised due to cultural or religious aspects due to sexual activity, especially men who have sex with men.  Also ensuring that cases are not missed in women and children due to misinformation about who is at risk. | The current study revealed the presence of knowledge gaps among healthcare workers regarding the infection. |
| Alvarez-Risco A. et al. | 2020 | 168 | Narrative (JCI) | Sierra leone | LMIC | Not specified | Pharmacist | Ebola virus | Collaboration at national level Support Health Care Worker and pharmacist education - national Development of IPC policy, guidance or recommendations - national Development of national treatment policy, recommendations or guidance Logistics and distribution of medicines or consumables - national Patient education or counselling - vaccination Symptom recognition and diagnosis Appropriate referral of patients |  |  |
| Andersson T. et al. | 2014 | 105 | Mixed methods (MMAT) | Sweden | HIC | Retail/community | Not specified | Not specific - infectious diseases | Supply of treatment to patients (OTC, sales or dispensing) Disease surveillance - use of pharmacy medicines sale data for early outbreak detection |  | The OTC sales data were only sensitive to the two largest outbreaks and revealed extreme values that did not correspond to any known outbreaks. |
| Anebonam U.P. and Ossai O. | 2016 | 169 | Case report (JCI) | Sierra leone | LMIC | Not specified | Pharmacist | Ebola virus | Pharmaceutical care of patients |  |  |
| Anonymous | 2015 | 130 | Other | Multiple | n/a | Industry | Not specified | Ebola virus | Vaccine clinical trials - organisational involvement Vaccine production - organisational involvement |  |  |
| Aremu T.O. et al. | 2021 | 131 | Expert opinion (JCI) | Multiple | n/a | Retail/community | Pharmacist | Not specific - pandemic, epidemic etc | Educate HCWs about antimicrobial stewardship National education for HCWs about antimicrobial stewardship Patient education or counselling - vaccination, reduce misinformation Patient education or counselling - prevention Advocate for access to vaccination - sharing with other countries Advocate for access to vaccination - unregistered patients Encourage national partnership with pharmaceutical companies Collaboration - to tackle health inequalities Review partnership and joint working to inform future response Point-of-care testing Support outbreak management - organisational/regional  Disease surveillance -recognition and reporting of unusual medication requests Lobby for better national public health measures | Sharing of vaccines across regions. Increased accessibility to vaccination in developed countries for underserved or illegal immigrants by advising to reduce personal information needed to get a vaccine. Increasing reach of information to "unreached" through targeted public communication. Collaboration with community and faith groups to reach diverse communities. |  |
| Asperti, F. et al. | 2023 | 99 | Quantitative Non-randomised (MMAT) | Italy | HIC | Multiple | Not specified | Not specific - pandemic, epidemic etc | Consider the use of novel solutions to provide usual pharmacy services Continued supply of medicines for long-term conditions | Increasing vulnerable patients quality of life and reducing risk from outbreak due to novel mechanisms of medicines supply |  |
| Auimekhakul T. et al. | 2024 | 179 | Policy/consensus guidelines (JCI) | Thailand | LMIC | Not specified | pharmacist | Not specific - infectious diseases | Disease surveillance -recognition and reporting of unusual medication requests Support outbreak management - organisational/regional  Involvement in emergency planning at organisational level Logistics and distribution of medicines or consumables - regional/organisational Post learning analysis and sharing - organisation |  |  |
| Azman, A.S. et al. | 2015 | 176 | Quantitative Non-randomised (MMAT) | Bangladesh | LMIC | Retail/community | Not specified | Cholera | Supply of treatment to patients (OTC, sales or dispensing) Disease surveillance - use of pharmacy medicines sale data for early outbreak detection |  | Barriers to surveillance in low income settings include lack of phone lines. |
| Badreldin H.A. et al. | 2020 | 148 | Expert opinion (JCI) | Saudi Arabia | HIC | Not specified | Pharmacist | Not specific - pandemic, epidemic etc | Development of national treatment policy, recommendations or guidance Involvement in emergency planning at organisational level Pharmaceutical care of patients Maintain organisational compliance - with guidance and recommendations Business continuity planning to allow continuation of pharmacy services Supply of treatment to patients (OTC, sales or dispensing) Patient education and advice - Symptom management Stay within law for supply of medicines Manage supply issues and medicines shortages Stock management of medicines and consumables - organisation Symptom recognition and diagnosis Vaccination Personnel management - Utilise retired or unemployed workforce Personnel management - national Enable expanded job roles at organisational level Develop, review or update legislation Undertake expanded job roles Changes in organisational ways of working to manage demand Public education - use of digital platforms Patient education and advice - symptoms Patient education or counselling - prevention Stock management of medicines and consumables - organisation Patient education and advice - Symptom management Public education - reduce misinformation Changes in organisational ways of working to manage demand Personnel management - Monitoring of staff illness Personnel management Development of IPC policy, guidance or recommendations - national Follow IPC recommendations Personnel management - risk assessment Changes in organisational ways of working to manage demand Personnel management - staff training Undertake self-education about pharmacy guidance and policies Personnel management Pharmaceutical care of patients Pharmaceutical care of patients Communication at national level Communication - support national communication methods Changes in organisational ways of working to manage demand Post learning analysis and sharing - organisation Contribute to clinical trials - individual Contribute to research - individual |  |  |
| Bartsch et al. | 2018 | 21 | Quantitative descriptive (MMAT) | United States of America | HIC | Retail/community | Not specified | Influenza | Vaccination | Increased accessibility to vaccination especially to those residing in medically underserved areas. | Although pharmacists are authorized to administer vaccinations, there may be policies which limit that authority. In the case of an epidemic, protocols may limit the ability for pharmacies to vaccinate. For example, state-imposed age restrictions limits pharmacists’ ability to vaccinate children. Furthermore, some insurance providers do not reimburse for pharmacist-provided services. |
| Bazzi, A.R. et al. | 2023 | 22 | Quantitative RCT (MMAT) | United States of America | HIC | Retail/community | Not specified | Human immunodeficiency virus (HIV) | Supply preventative medicines or measures to patients - HIV PREP Pharmaceutical care of patients Adherence support for treatment | Support for patients who inject drugs suffering homelessness to access treatment and prevention | Storage - those suffering homelessness struggled to safely store, prepare and access medicines. Continuity - difficulty maintaining consistent access to refills due to unstable living conditions and irregular clinic attendance; system navigation change - participants found traditional pharmacy systems difficult to navigate (in relation to participants not having medical insurance. |
| Bazzi, A.R. et al. | 2023 | 23 | Qualitative study (MMAT) | United States of America | HIC | Retail/community | Not specified | Human immunodeficiency virus (HIV) | Supply of treatment to patients (OTC, sales or dispensing) Disease surveillance - use of pharmacy data |  |  |
| Bell, S.A. et al. | 2017 | 165 | Qualitative study (MMAT) | Liberia | LMIC | Not specified | Pharmacy dispenser | Ebola virus | Contribute to research - individual Respond to outbreak |  |  |
| Berthelemy S. | 2015 | 79 | Expert opinion (JCI) | France | HIC | Not specified | Pharmacist | Influenza | Patient education and advice - Symptom management Patient education or counselling - prevention Patient education or counselling - vaccination Vaccination - recognition of at risk patients to prioritise for vaccination |  |  |
| Bertomeu Sanchez, J.R. et al. | 2018 | 84 | Case report (JCI) | Spain | HIC | Not specified | Pharmacist | Poisoning | Symptom recognition and diagnosis Part of outbreak response Multi-Disciplinary Team Patient education or counselling - prevention |  |  |
| Bora, A. et al. | 2022 | 172 | Quantitative Non-randomised (MMAT) | India | LMIC | Hospital | Not specified | Not specific - pandemic, epidemic etc | Logistics and distribution of medicines or consumables - regional/organisational |  |  |
| Bouza E. et al. | 2023 | 85 | Policy/consensus guidelines (JCI) | Spain | HIC | Long term care setting | Not specified | Not specific - infectious diseases | Pharmaceutical care of patients Disease surveillance - recognition and reporting of symptoms in NH residents Development of organisational treatment policy or guidance | Targeted support to Nursing Homes |  |
| Buicu, C.-F. et al. | 2018 | 103 | Narrative (JCI) | Romania | HIC | Retail/community | Not specified | Not specific - infectious diseases | Vaccination Patient education or counselling - vaccination | Increased accessibility to vaccination  Targeted information to at-risk groups. |  |
| Cai, W. et al. | 2019 | 108 | Quantitative descriptive (MMAT) | China | LMIC | Public health workforce | Pharmacist | Not specific - infectious diseases | Leadership role at national level Part of outbreak response Multi-Disciplinary Team |  | Need better incentives to attract public health workers in some areas. |
| Calabria C.L. et al. | 2014 | 24 | Narrative (JCI) | United States of America | HIC | Hospital | Pharmacist | Scabies | Supply of treatment to patients (OTC, sales or dispensing) Changes in organisational ways of working to manage demand Patient education or counselling - prevention Patient education and advice - use of treatment |  |  |
| Chatterjee, S. et al. | 2025 | 173 | Quantitative Non-randomised (MMAT) | India | LMIC | Retail/community | Not specified | Acute hemorrhagic conjunctivitis | Patient education and advice - Symptom management | Increased accessibility to treatment for symptoms. | Most pharmacies are staffed by non-qualified personnel, the practice of dispensing drugs without a prescription or providing treatment for minor ailments is fraught with risks of worsening an uncomplicated medical condition or delaying the institution of appropriate therapy. |
| Chen D. et al. | 2020 | 109 | Qualitative study (MMAT) | China | LMIC | Retail/community | Pharmacist | Not specific - pandemic, epidemic etc | Continued supply of medicines for long-term conditions Manage medicine and consumable supply issues - organisation | Continued access to medicines for long-term conditions. | Limited supply of regular medications. |
| Chukwu O.A. | 2018 | 162 | Other | Nigeria | LMIC | Not specified | Pharmacist | Not specific - infectious diseases | Knowledge of Health Technology Appraisals |  | 93% of pharmacists not aware of Health Technology Appraisals; there is increasing need to raise awareness on the importance of Health Technology Appraisals in healthcare decision-making; strengthen Health Technology Appraisals capacity |
| Connor, S.E. et al. | 2022 | 132 | Expert opinion (JCI) | Multiple | n/a | Retail/community | Pharmacist | Not specific - pandemic, epidemic etc | Continued supply of medicines for long-term conditions Manage medicine and consumable supply issues - organisation Forecasting organisational recovery needs Logistics and distribution of medicines or consumables - regional/organisational | Increased access to medicines in an emergency Empowerment of local communities |  |
| Czech, M. et al. | 2020 | 93 | Systematic review (JCI) | Poland | HIC | Retail/community | Pharmacist | Influenza | Vaccination Patient education or counselling - vaccination | Increased accessibility to vaccination | Barriers that may arise from patients should also be emphasized. Anti-vaccination movements can be of particular importance. Patients’ concerns about vaccination, such as pain or fear of side effects, may also be significant |
| Datkhayev U. et al. | 2016 | 100 | Quantitative descriptive (MMAT) | Kazakhstan | LMIC | Retail/community | Pharmacist | Influenza | Business continuity planning to allow continuation of pharmacy services |  |  |
| DeBenedette V. | 2016 | 25 | Other | United States of America | HIC | Not specified | Pharmacist | Zika | Patient education and advice - disease |  |  |
| Delcher C. et al. | 2024 | 26 | Quantitative descriptive (MMAT) | United States of America | HIC | Retail/community | Not specified | Human immunodeficiency virus (HIV) | Disease surveillance - use of pharmacy data for pharmacovigilance |  | Pharmacy transaction data did not trigger public health responses early enough to prevent injection-related harms. Although data existed, they weren’t effectively used to detect misuse trends or intervene at the pharmacy level. |
| Demler T.L. | 2015 | 27 | Narrative (JCI) | United States of America | HIC | Not specified | Pharmacist | Enterovirus D68 | Patient education and advice - Symptom management Public education - disease Triage and appropriate referral of patients |  |  |
| Denninger, J. et al. | 2023 | 28 | Narrative (JCI) | United States of America | HIC | Retail/community | Pharmacy student | Mpox or Monkeypox | Vaccination Patient education or counselling - vaccination and prevention Undertake expanded job roles | Increased accessibility to vaccination. | Pharmacy student interns can successfully vaccinate using the intradermal route of administration, counsel patients, and perform many other functions that can fill any gaps in care during this public health outbreak. New Mexico have a unique setting where the law allow and pharmacists already train to administer intradermal vaccine but needs to expand to include pharmacy students. |
| Ding, Y. et al. | 2015 | 110 | Quantitative descriptive (MMAT) | China | LMIC | Retail/community | Not specified | Not specific - infectious diseases | Supply of treatment to patients (OTC, sales or dispensing) Disease surveillance - use of pharmacy medicines sale data for early outbreak detection |  |  |
| Doan, D.A. et al. | 2024 | 123 | Quantitative descriptive (MMAT) | Vietnam | LMIC | Higher Education Institutions | Pharmacy student | Mpox or Monkeypox | Public education - disease Respond to outbreak |  | Students’ knowledge about mpox was relatively poor, while their attitudes were comparatively positive. |
| Dong, X. et al. | 2017 | 111 | Quantitative descriptive (MMAT) | China | LMIC | Retail/community | Not specified | Influenza | Supply of treatment to patients (OTC, sales or dispensing) Disease surveillance - use of pharmacy medicines sale data for early outbreak detection |  |  |
| Dowling-McClay K. et al. | 2021 | 29 | Qualitative study (MMAT) | United States of America | HIC | Retail/community | Pharmacist | Human immunodeficiency virus (HIV) and Hepatitis C (HVC) | Supply preventative medicines or measures to patients - clean syringes Patient education or counselling - prevention Triage and appropriate referral of patients | Use pharmacists’ trusted role and extended hours to make prevention services more available in rural areas for PWID | Personal discomfort or moral concerns with serving people who inject drugs, lack of training and awareness: many felt unprepared to provide HIV/HCV-related services due to limited education or guidance, legal confusion around non-prescription syringe sales and harm reduction activities created uncertainty and hesitancy, time constraints and lack of support staff, fear of community pushback: Pharmacists feared negative reactions from customers or community members if they offered harm reduction services. |
| Elsayed A.A. et al. | 2023 | 153 | Quantitative Non-randomised (MMAT) | Egypt | LMIC | Multiple | Pharmacist | Mpox or Monkeypox | Patient education and advice - disease Public education - reduce misinformation Public education - disease |  | Gap identified in this research is the knowledge about ways of transmission, prevention, and prophylaxis. It is very noticeable that all three groups have poor knowledge about vaccination available and other preventive measures |
| Evans A. et al. | 2016 | 89 | Quantitative descriptive (MMAT) | United Kingdom | HIC | Not specified | Pharmacist | Not specific - infectious diseases | Undertake self education - communicable disease |  |  |
| Fadanelli M. et al. | 2022 | 30 | Qualitative study (MMAT) | United States of America | HIC | Retail/community | Pharmacist | Human immunodeficiency virus (HIV) and Hepatitis C (HVC) | Supply preventative medicines or measures to patients - clean syringes | Increased accessibility to harm reduction for PWID | Many pharmacists stigmatised people who inject drugs, viewing syringe access as enabling drug use |
| Fava, J.P. et al. | 2020 | 31 | Narrative (JCI) | United States of America | HIC | Retail/community | Pharmacist | Not specific - infectious diseases | Vaccination | Increased accessibility to vaccination. |  |
| Finnes, H.D. et al | 2023 | 32 | Policy/consensus guidelines (JCI) | United States of America | HIC | Hospital | Multiple | Not specific - pandemic, epidemic etc | Clinical trials - changes in ways of working Clinical trial - supply of investigational medicines to patients Clinical trials - organisational involvement Patient education and advice via digital platforms | Set up of virtual systems to support clinical trials may overcome recognised barriers to clinical trial accrual such as socioeconomic disparities and demographics. | Lack of transportation, travel costs, lack of childcare, socioeconomic disparities and demographics, lack of knowledge, fear, need for face to face meetings are barriers to patients taking part in research trials - more focus on the institution than the patient. Barriers to virtual working: scheduling calls around patient availability. |
| Fischer L.J. et al. | 2022 | 33 | Expert opinion (JCI) | United States of America | HIC | Not specified | Pharmacist | Not specific - pandemic, epidemic etc | National education for pharmacy professionals about vaccination Vaccination | Increased accessibility to vaccination on islands |  |
| Fitzgerald T.J. et al. | 2016 | 34 | Qualitative study (MMAT) | United States of America | HIC | Retail/community | Multiple | Influenza | Vaccination Involvement in national vaccination program |  | Incomplete identification of points of contact, inconsistent processes for recruiting pharmacists as pandemic vaccinators, lack of clarity on the responsibility for outreach to and enrolment of pharmacists as pandemic vaccinators, inconsistencies in pandemic vaccine allocation and distribution plans to pharmacies, limited information on pharmacy patient populations and storage and handling capacity, lack of training for pharmacists as vaccinators, plans for late-stage involvement of pharmacy reducing capacity and potential. |
| Fokam, J. et al. | 2015 | 170 | Quantitative descriptive (MMAT) | Cameroon | LMIC | Primary care | Not specified | Human immunodeficiency virus (HIV) | Supply of treatment to patients (OTC, sales or dispensing) Disease surveillance - use of pharmacy data |  |  |
| Fujimura, S. | 2020 | 117 | Other | Japan | HIC | Retail/community | Pharmacist | Not specific - pandemic, epidemic etc | Follow IPC policy, guidance or recommendations Patient education and advice Collaboration - across sectors Support IPC in care homes |  | Access to specialised training, constraints of the physical environment to support proper infection control measures |
| Garibaldi, B.T. et al. | 2016 | 35 | Narrative (JCI) | United States of America | HIC | Hospital | Not specified | Ebola virus | Part of outbreak response Multi-Disciplinary Team |  |  |
| Gautam, K. et al. | 2024 | 120 | Quantitative descriptive (MMAT) | Malaysia | LMIC | Retail/community | Pharmacist | Human immunodeficiency virus (HIV) | Patient education and advice via digital platforms |  | Challenges with patient engagement and sustained use of digital platforms, need for adequate training and support for pharmacists to use digital tools effectively, technology access and literacy barriers among some patient populations. |
| Gimbar P. et al. | 2018 | 36 | Other | United States of America | HIC | Hospital | Pharmacist | Poisoning | Pharmaceutical care of patients Manage supply issues and medicines shortages Respond to outbreak Supply of treatment to patients (OTC, sales or dispensing) Long-term follow up and management of lasting effects of outbreak | Set up of telephone clinic for care of patients requiring long-term follow up allows adaptability to patient's needs, including in urban or rural areas. |  |
| Goncalo S.P. | 2016 | 76 | Narrative (JCI) | Brazil | LMIC | Multiple | Pharmacist | Zika | Symptom recognition and diagnosis Disease surveillance - reporting of cases Patient education or counselling - prevention Public education - reduce misinformation Patient education and advice Support Health Care Worker and pharmacist education - national Development of national treatment policy, recommendations or guidance Develop national public education resources and campaigns |  |  |
| Gouglas D. et al. | 2018 | 133 | Quantitative descriptive (MMAT) | Multiple | n/a | Industry | Not specified | Not specific - infectious diseases | Vaccine development and production - national |  |  |
| Grimshaw C. et al. | 2020 | 90 | Quantitative descriptive (MMAT) | United Kingdom | HIC | Retail/community | Pharmacist | Human immunodeficiency virus (HIV) | Preventative medicine adherence support - HIV PREP Collaboration - across sectors | Community pharmacies key players in reaching treatment and prevention to vulnerable groups | Need for tailored service modifications to overcome barriers in case finding, initiation, and adherence. Challenges with engagement, such as patients becoming untraceable or entering rehab/prison affecting continuity |
| Guerci J. et al. | 2020 | 37 | Expert opinion (JCI) | United States of America | HIC | Multiple | Multiple | Not specific - infectious diseases | Patient education or counselling - prevention Advocate for access to vaccination Vaccination Assessing vaccination status Patient education or counselling - vaccination Maintain organisational compliance - with Infection, Prevention, and Control guidance Changes in organisational ways of working to manage demand Patient education and advice - Symptom management Continued supply of medicines for long-term conditions Point-of-care testing Maintain professional competence Clinical trials - advocate Involvement in patient identification, notification, and contract tracing Patient education and advice - Symptom management Point-of-care testing Undertake expanded job roles |  | While pharmacists have extensive history with providing outpatient immunizations, regulations have historically placed limits on the types of vaccines and age groups that can receive them. Also regulation and physician and parental attitudes. |
| Gunta B. et al. | 2021 | 174 | Other | India | LMIC | Primary care | Pharmacist | Rubella | Logistics and distribution of medicines or consumables - regional/organisational |  | Shortage of vaccine |
| Gupta N. et al. | 2014 | 38 | Case report (JCI) | United States of America | HIC | Compounding pharmacy or centre | Multiple | Contamination of pharmaceutical product | Follow aseptic policy or guidance when making up pharmaceutical products - individual |  |  |
| Hall D.L. | 2017 | 39 | Narrative (JCI) | United States of America | HIC | Retail/community | Pharmacist | Influenza | Integration of pharmacies into national emergency planning Development of vaccination policy, guidance or recommendations Maintain professional competence Involvement in emergency planning at organisational level Vaccination |  | Gaps in including pharmacists in planning and preparation, how pharmacists were engaged and enrolled as vaccine providers; vaccine allocation and distribution; and utilization of formal agreements or memoranda of understanding. Also training and education of pharmacists. |
| Heudtlass P. et al. | 2020 | 102 | Other | Portugal | HIC | Retail/community | Not specified | Influenza | Supply of treatment to patients (OTC, sales or dispensing) Disease surveillance - use of pharmacy medicines sale data for early outbreak detection |  |  |
| Hicks, R.W. and Blackwell, C. | 2023 | 40 | Narrative (JCI) | United States of America | HIC | Primary care | Pharmacist | Mpox or Monkeypox | Vaccine distribution - organisation | Increased accessibility of vaccination to high-risk groups |  |
| Huang, X.H. et al. | 2024 | 41 | Quantitative descriptive (MMAT) | United States of America | HIC | Prisons | Pharmacist | Human immunodeficiency virus (HIV) | Long-term follow up and management of lasting effects of outbreak Educate Health Care Workers about the disease Supply preventative medicines or measures to patients - HIV PREP |  | Challenges to program implementation include adequate staffing |
| Jabeen R. et al. | 2021 | 151 | Narrative (JCI) | Pakistan | LMIC | Multiple | Multiple | Antimicrobial resistance (AMR) | Antimicrobial stewardship Support organisational antimicrobial stewardship Support national antimicrobial stewardship |  | Dispensing time is inadequate for good dispensing, communication with patients is poor. Preparation of prescriptions by dispensers is often unhygienic, prone to mistakes and one in 5 prescriptions is dispensed without validation. Labelling and record keeping is poor. Utilisation of fake, poor quality and unreliable antimicrobials and predisposition to more expensive broad spectrum antibiotics. Unregulated pharmacies and lack of coordination among relevant professionals, ratio of 1:2000 pharmacists per population as recommended by WHO- in Pakistan it is 0.9 or 100000 |
| Jacquerioz Bausch, F.A. et al. | 2018 | 134 | Narrative (JCI) | Multiple | n/a | Hospital | Pharmacist | Ebola virus | Production of preventative measures - alcohol based hand sanitiser | Local manufacture of preventative consumables to target urban and rural settings | Sustainability and long term technical support |
| Jafari, K. et al. | 2016 | 154 | Quantitative descriptive (MMAT) | Iran | LMIC | Retail/community | Not specified | Influenza | Supply of treatment to patients (OTC, sales or dispensing) Disease surveillance - use of pharmacy medicines sale data for early outbreak detection |  | Issues with usual pattern of use of the data |
| Jalloh, M.F. et al. | 2019 | 177 | Qualitative study (MMAT) | Bangladesh | LMIC | Retail/community | Pharmacist | Diphtheria | Vaccination Patient education and advice - Symptom management Patient education or counselling - vaccination | Increased accessibility of vaccination to displaced people |  |
| Jambrina, A.M et al. | 2022 | 86 | Narrative (JCI) | Spain | HIC | Retail/community | Pharmacist | Not specific - pandemic, epidemic etc | Supply of treatment to patients (OTC, sales or dispensing) Disease surveillance - use of pharmacy medicines sale data for early outbreak detection |  |  |
| James P.B. et al. | 2016 | 135 | Expert opinion (JCI) | Multiple | n/a | Multiple | Multiple | Ebola virus | Disease surveillance - reporting of cases Collaboration - to tackle health inequalities Vaccine distribution Vaccine distribution - organisation Pharmaceutical care of patients Contribute to clinical trials - individual Quality assurance of new treatments Quality assurance of new vaccines Leadership role at organisational level Leadership role at national level Collaboration at national level First point of patient contact Symptom recognition and diagnosis Triage and appropriate referral of patients Patient education or counselling - prevention Patient education and advice - treatment Public education - reduce misinformation Vaccination Development of organisational emergency planning policies and guidance Pharmaceutical care of patients Development of organisational treatment policy or guidance |  | The local health authorities and the international communities were initially ill-prepared to effectively respond to an outbreak of such magnitude. Poor knowledge about the disease among most healthcare professionals including pharmacists and the populace combined with a dysfunctional health system due to the prolonged civil conflicts and an initial piece-meal response from the international community led to the spread of Ebola |
| Jiang H. et al. | 2014 | 112 | Other | China | LMIC | Retail/community | Not specified | Not specific - infectious diseases | Supply of treatment to patients (OTC, sales or dispensing) Disease surveillance - use of pharmacy medicines sale data for early outbreak detection | Able monitor sales of medicines in rural areas | Training were still required to improve their knowledge of syndromic surveillance and ability of using a computer. |
| Jinadu, K.A. et al. | 2018 | 163 | Qualitative study (MMAT) | Nigeria | LMIC | Primary care | Pharmacy technician | Not specific - pandemic, epidemic etc | Knowledge of how to report key diseases |  | Barriers included poor knowledge of specific surveillance methods, limited training and inadequate supervision, lack of feedback from higher levels of the surveillance system. |
| Karakilias, C. | 2021 | 95 | Quantitative descriptive (MMAT) | Turkey | LMIC | Retail/community | Pharmacist | Smallpox | Knowledge of vaccination policy, guidance or recommendations Vaccination Vaccine distribution - organisation Vaccine distribution - national | Increased accessibility to vaccination in rural areas | Resistance to vaccination among certain populations. Logistical challenges in vaccine distribution, especially in remote areas. Need for trained medical personnel to administer vaccines effectively. |
| Kawachi A. et al. | 2017 | 118 | Case report (JCI) | Japan | HIC | Retail/community | Pharmacist | Influenza | Symptom recognition and diagnosis Point-of-care testing Long-term follow up and management of lasting effects of outbreak | |  |
| Keats, K. et al. | 2022 | 42 | Narrative (JCI) | United States of America | HIC | Hospital | Pharmacist | Not specific - pandemic, epidemic etc | Provide education for pharmacy professionals in organisation - emergency planning Undertake self education - emergency planning |  | Limited prior experience among residents in disaster preparedness |
| Khan, M.S. et al. | 2017 | 178 | Qualitative study (MMAT) | Myanmar | LMIC | multiple | Pharmacist | Tuberculosis | Supply of treatment to patients (OTC, sales or dispensing) Symptom recognition and diagnosis Pharmaceutical management of long-term conditions Follow treatment guidance | The study notes gender disparities in the assignment of treatment supporters, with men less likely to have one, indicating potential gender-based health inequalities | High reliance on private healthcare providers, which may not follow standardized treatment protocols, lack of treatment supporters for a significant proportion of patients, limited patient knowledge about the consequences of missing treatment, patient complexities. |
| King, W.B. et al. | 2024 | 43 | Expert opinion (JCI) | United States of America | HIC | Hospital | Not specified | Ebola virus | Review and update organisational protocol, policies, and guidelines Follow IPC recommendations | |  |
| Lapian B. et al. | 2022 | 44 | Case report (JCI) | United States of America | HIC | Hospital | Pharmacist | Measles | Part of outbreak response Multi-Disciplinary Team Pharmaceutical care of patients | Increased vaccination accessibility for refugees | Consideration and evaluation of identification during massive refugee evacuation and vaccination. |
| Lenhard-Schramm, N. | 2020 | 136 | Expert opinion (JCI) | Multiple | n/a | Multiple | Pharmacist | Not specific - pandemic, epidemic etc | Supply of treatment to patients (OTC, sales or dispensing) Vaccine dispensing Public education Collaboration at national level Communication - with wider teams involved in response Logistics and distribution of medicines or consumables - regional/organisational Integration of pharmacies into national emergency planning Follow IPC policy, guidance or recommendations | The article discusses the role of pharmacies in improving access to healthcare services, particularly in underserved communities, during epidemics. | Challenges in supply chain management during epidemics. Need for clear communication channels between pharmacies and health authorities. |
| Leung E. and Kwee F. | 2019 | 45 | Quantitative descriptive (MMAT) | United States of America | HIC | Hospital | Pharmacist | Clostridium difficile | Part of outbreak response Multi-Disciplinary Team |  | Challenges in changing prescribing behaviour |
| Liang, Y. et al. | 2017 | 113 | Narrative (JCI) | China | LMIC | Industry | Not specified | Influenza | Pharmaceutical industry role in developing treatments |  | The article emphasizes the challenges in antiviral drug development, including drug resistance and the need for novel therapeutic targets. |
| Lisenby K.M. et al. | 2021 | 46 | Narrative (JCI) | United States of America | HIC | Retail/community | Pharmacist | Measles | Patient education or counselling - vaccination Vaccination Involvement in public health campaigns Patient education or counselling - vaccination, reduce misinformation Collaboration - with wider teams involved in response | Increased accessibility to vaccination for vulnerable groups | Misinformation and distrust in vaccines, limited pharmacist authority in some regions, need for better integration with public health systems. |
| Liu Y. et al. | 2014 | 114 | Quantitative descriptive (MMAT) | China | LMIC | Hospital | Not specified | Multi-drug resistant organism outbreak | Follow IPC policy, guidance or recommendations |  | Importance of rapid identification and response , environmental reservoirs can be overlooked, need for strict adherence to infection control protocols. |
| Lutwick, L. | 2014 | 47 | Expert opinion (JCI) | United States of America | HIC | Compounding pharmacy or centre | Pharmacist | Contamination of pharmaceutical product | Recall of contaminated pharmaceutical products Follow aseptic policy or guidance when making up pharmaceutical products - organisation |  |  |
| Malaeb D. et al. | 2023 | 158 | Quantitative descriptive (MMAT) | Lebanon | LMIC | Hospital | Pharmacist | Mpox or Monkeypox | Patient education and advice - treatment Patient education or counselling - prevention Public education - reduce misinformation Knowledge about the disease/outbreak |  | The study found significant gaps in knowledge about monkeypox among healthcare workers and a notable prevalence of conspiracy beliefs, which could hinder effective public health responses. |
| Marshall L. et al. | 2018 | 48 | Policy/consensus guidelines (JCI) | United States of America | HIC | Long term care setting | Not specified | Influenza | Supply of treatment to patients (OTC, sales or dispensing) Vaccination Logistics and distribution of medicines or consumables - regional/organisational | Increased accessibility to vaccination in long-term care facilities Improved outbreak management in long-term care facilities | Delayed recognition of outbreaks, low vaccination uptake, limited antiviral availability, lack of staff knowledge and challenges in isolating residents. |
| Masood, S. et al. | 2023 | 152 | Quantitative descriptive (MMAT) | Pakistan | LMIC | Higher Education Institutions | Pharmacy student | Mpox or Monkeypox | Undertake self education - communicable disease |  | The study found moderate knowledge levels among students, with significant gaps in understanding of transmission routes and prevention. Barriers include lack of formal education or training on monkeypox in the curriculum. |
| Mathias, C. | 2015 | 49 | Narrative (JCI) | United States of America | HIC | Higher Education Institutions | Pharmacy student | Not specific - infectious diseases | Provide education for pharmacy professionals in organisation - emergency planning |  | A challenge noted was ensuring consistent participation and preparation for peer-led discussions during education. |
| McCarthy, M.W. and Walsh, T.J. | 2017 | 50 | Narrative (JCI) | United States of America | HIC | Compounding pharmacy or centre | Not specified | Contamination of pharmaceutical product | Follow aseptic policy or guidance when making up pharmaceutical products - organisation |  |  |
| McCree D.H. et al. | 2020 | 51 | Expert opinion (JCI) | United States of America | HIC | Not specified | Pharmacist | Human immunodeficiency virus (HIV) | Supply of treatment to patients (OTC, sales or dispensing) Supply preventative medicines or measures to patients - HIV PREP Point-of-care testing Adherence support for treatment Long-term follow up and management of lasting effects of outbreak Supply preventative medicines or measures to patients - clean syringes |  | Workforce capacity issues, gaps in reimbursement mechanisms, regulatory or policy restrictions, need for training and integration within public health systems. |
| Minhaj F.S. et al. | 2022 | 52 | Expert opinion (JCI) | United States of America | HIC | Public health workforce | Pharmacist | Not specific - infectious diseases | Disease surveillance - involvement in evaluation of national surveillance systems Collaborate on national reports about learning from outbreaks Pharmaceutical product source identification Part of Multi-disciplinary Team that leads on national preparedness Development of national treatment policy, recommendations or guidance Clinical trials - design and implementation  Development of national treatment policy, recommendations or guidance Part of national Multi-disciplinary Team for outbreak response Increase awareness of national opportunities for pharmacy professionals in public health roles |  | Limited awareness among pharmacists about opportunities in public health programs, and historically low participation of pharmacists. |
| Mirtella, D. et al. | 2021 | 80 | Case report (JCI) | France | HIC | Hospital | Pharmacist | Contamination of pharmaceutical product | Pharmaceutical product source investigation Development of aseptic policy or guidance for pharmaceutical products - organisational |  | Initial difficulty in identifying the contamination source. |
| Moehring R.W. et al. | 2014 | 53 | Case report (JCI) | United States of America | HIC | Compounding pharmacy or centre | Pharmacist | Contamination of pharmaceutical product | Follow aseptic policy or guidance when making up pharmaceutical products - individual Follow aseptic policy or guidance when making up pharmaceutical products - organisation |  | Barriers included delayed recognition of the outbreak and challenges in tracing the source |
| Moheet, A.M. et al. | 2020 | 54 | Policy/consensus guidelines (JCI) | United States of America | HIC | Hospital | Pharmacist | Not specific - pandemic, epidemic etc | Business continuity planning to allow continuation of pharmacy services Development of organisational policy or guidance - emergency response Pharmaceutical management of long-term conditions Part of outbreak response Multi-Disciplinary Team |  | Barriers include limited ICU beds, ventilators, and trained staff. |
| Moreno-Perez, D. et al. | 2014 | 87 | Policy/consensus guidelines (JCI) | Spain | HIC | Primary care | Not specified | Not specific - pandemic, epidemic etc | Vaccine dispensing Public education - prevention and vaccination |  | Regional disparities in vaccine funding and access, and the need for improved public awareness and adherence. |
| Mumcuoglu, K.Y. et al. | 2021 | 137 | Policy/consensus guidelines (JCI) | Multiple | n/a | Retail/community | Pharmacist | Headlice | Public education - disease Supply of treatment to patients (OTC, sales or dispensing) | Pharmacist role in addressing disparities in access to effective treatments and the impact of socioeconomic status on infestation rates and treatment adherence. | Treatment resistance, misuse of products, lack of awareness, and inconsistent school policies. |
| Nadendla, R.R. et al. | 2024 | 91 | Expert opinion (JCI) | United Kingdom | HIC | Retail/community | Pharmacist | Not specific - pandemic, epidemic etc | Vaccination Public education Support patient level antimicrobial stewardship Adherence support for treatment Point-of-care testing | Pharmacists improving access to care in underserved or rural communities. | Barriers may include regulatory limitations, lack of public awareness, and underutilization of pharmacists. |
| Narayanan N. et al. | 2018 | 55 | Narrative (JCI) | United States of America | HIC | Hospital | Pharmacist | Biohazard | Pharmaceutical care of patients Pharmaceutical care of patients Development of organisational policy or guidance - treatment Logistics and distribution of medicines or consumables - regional/organisational Public education Part of outbreak response Multi-Disciplinary Team | Biological threats can disproportionately affect vulnerable populations, and pharmacists can help mitigate disparities through community outreach and equitable access to treatment. | Lack of formal training in disaster preparedness for pharmacists, limited integration into emergency planning teams, and inconsistent access to medical countermeasures. |
| Nassar, R.I. et al. | 2024 | 159 | Quantitative descriptive (MMAT) | United Arab Emirates | HIC | Multiple | Pharmacist | Mpox or Monkeypox | Patient education or counselling - prevention Symptom recognition and diagnosis Patient education and advice - disease |  | The study found moderate knowledge levels and identified gaps in understanding of monkeypox transmission and prevention. Barriers included lack of formal training and limited exposure to emerging infectious disease content in curricula. |
| Nexon, M. | 2024 | 138 | Expert opinion (JCI) | Multiple | n/a | Retail/community | Pharmacist | Respiratory Syncytial Virus | Public education - vaccination Patient education or counselling - prevention Vaccination |  | Limited vaccine access, lack of pharmacist prescribing authority in some regions, public awareness. |
| Nhi, L.T.Q. et al. | 2018 | 124 | Mixed methods (MMAT) | Vietnam | LMIC | Hospital | Pharmacist | Paediatric diarrhoeal disease | Supply of treatment to patients (OTC, sales or dispensing) |  | Need for better prescribing guidelines and healthcare worker and patient/carer education. |
| Nolan, A. | 2014 | 56 | Policy/consensus guidelines (JCI) | United States of America | HIC | Compounding pharmacy or centre | Pharmacist | Contamination of pharmaceutical product | Follow aseptic policy or guidance when making up pharmaceutical products - individual |  | Balancing access to compounded drugs with safety, state vs. federal jurisdiction |
| Olivier H. et al. | 2015 | 166 | Quantitative descriptive (MMAT) | Liberia | LMIC | Hospital | Pharmacist | Ebola virus | Production of preventative measures - alcohol based hand sanitiser Educate HCWs on how to produce preventative measures - alcohol based hand sanitiser Supply preventative medicines or measures to patients - alcohol based hand sanitiser | Highlighted the need for equitable access to IPC resources in low-resource settings. | Lack of sustained funding, limited raw material supply, need for ongoing training. |
| O'Neil, M.J. et al. | 2024 | 57 | Quantitative descriptive (MMAT) | United States of America | HIC | Hospital | Pharmacist | Mpox or Monkeypox | Supply of treatment to patients (OTC, sales or dispensing) Logistics and distribution of medicines or consumables - regional/organisational Patient education and advice - treatment | can support equitable access especially for LGBTQ+ and minority communities disproportionately affected by mpox | Regulatory hurdles, limited supply, need for rapid education of providers and lack of centralised coordination. |
| Ouoba, K. et al. | 2022 | 139 | Narrative (JCI) | Multiple | n/a | Not specified | Pharmacist | Not specific - pandemic, epidemic etc | Involvement in public health campaigns | The article discusses the colonial health system's aim to protect both colonizers and indigenous populations, implying a dual focus that may have led to disparities in healthcare delivery and access. |  |
| Paquette, C.E. and Pollini, R.A. | 2018 | 58 | Systematic review (JCI) | United States of America | HIC | Not specified | Not specified | Human immunodeficiency virus (HIV) | Supply preventative medicines or measures to patients - clean syringes |  | Availability varied by jurisdiction and pharmacy discretion, testing and harm reduction services were largely absent from nonurban settings |
| Parks, K.C. et al. | 2015 | 59 | Expert opinion (JCI) | United States of America | HIC | Compounding pharmacy or centre | Pharmacist | Contamination of pharmaceutical product | Follow aseptic policy or guidance when making up pharmaceutical products - organisation Follow aseptic policy or guidance when making up pharmaceutical products - organisation Pharmaceutical product source investigation |  |  |
| Pashkov, V. et al. | 2022 | 107 | Qualitative study (MMAT) | Ukraine | LMIC | Retail/community | Pharmacist | Not specific - pandemic, epidemic etc | Advocate/lobby for patients Manage medicine and consumable supply issues Management of national drug costs Develop, review or update legislation | The article highlights the role of pharmacists in managing price gouging during emergencies, which can exacerbate health inequalities by limiting access to affordable medicines for vulnerable populations. |  |
| Piccolo, A.J.L. et al. | 2023 | 60 | Expert opinion (JCI) | United States of America | HIC | Hospital | Not specified | Mpox or Monkeypox | Part of Infection, Prevention, and Control Multi-disciplinary Team | Increased accessibility to vaccination | Early challenges included health inequities, inadequate access, and lack of vaccine uptake. |
| Pierce, W.F. et al. | 2017 | 140 | Policy/consensus guidelines (JCI) | Multiple | n/a | Hospital | Not specified | Ebola virus | Stock management of medicines and consumables - organisation | The article emphasizes the challenges faced in resource-limited settings, highlighting disparities in access to essential medications during EVD outbreaks and the role of pharmacists in maintaining stock.. | A significant proportion of medications listed on the Emergency Medicines List applicable for this infection, indicating a need to tailor to specific outbreak scenarios. |
| Pierson J.F. et al. | 2017 | 167 | Narrative (JCI) | Liberia | LMIC | Research pharmacy | Pharmacist | Ebola virus | Vaccine clinical trial - establishing a research pharmacy Vaccine dispensing Vaccine clinical trials - training of staff | Lack of research infrastructure in Liberia and the need for capacity building to address health disparities. | Challenges included establishing research infrastructure in a resource-limited setting and training local pharmacists in research protocols. |
| Plans Rubio P. et al. | 2022 | 88 | Quantitative descriptive (MMAT) | Spain | HIC | Retail/community | Pharmacist | Influenza | Symptom recognition and diagnosis Disease surveillance - reporting of cases Disease surveillance - use of pharmacy medicines sale data for early outbreak detection |  |  |
| Qadah, T. | 2023 | 149 | Quantitative descriptive (MMAT) | Saudi Arabia | HIC | Not specified | Pharmacist | Mpox or Monkeypox | Knowledge about the disease/outbreak |  | The study found that healthcare workers in Saudi Arabia had an inadequate understanding of monkeypox, highlighting the need for improved education and training |
| Rajiah, K. et al. | 2019 | 121 | Quantitative descriptive (MMAT) | Malaysia | LMIC | Retail/community | Pharmacist | Zika | Knowledge about the disease/outbreak |  | Community pharmacists exhibited lower levels of preparedness and perceived response capabilities compared to general practitioners. Lack of targeted training and awareness programs for pharmacists was identified as a barrier |
| Ramsey T.D. et al. | 2015 | 78 | Expert opinion (JCI) | Canada | HIC | Hospital | Pharmacist | Ebola virus | Logistics and distribution of medicines or consumables - regional/organisational Follow IPC recommendations Logistics and distribution of medicines or consumables - regional/organisational Development of organisational policy or guidance Supply of treatment to patients (OTC, sales or dispensing) Follow IPC policy, guidance or recommendations Development of organisational policy or guidance - Infection, prevention, and control |  | Challenges with documentation and medication disposal in isolation settings. |
| Rasaq Kayode O. and Afeez Babatunde O. | 2021 | 164 | Expert opinion (JCI) | Nigeria | LMIC | Retail/community | Pharmacist | Human immunodeficiency virus (HIV) | Point-of-care testing Preventative medicine adherence support - HIV PREP Supply preventative medicines or measures to patients - HIV PREP Supply of treatment to patients (OTC, sales or dispensing) Patient education and advice - treatment |  | Lack of policy frameworks to integrate pharmacists formally into HIV service delivery, training gaps in handling new technologies like long-acting injectables, infrastructure limitations in community pharmacy settings, stigma and patient privacy concerns in community-based settings. |
| Robinson, R. et al. | 2023 | 61 | Mixed methods (MMAT) | United States of America | HIC | Retail/community | Pharmacist | Not specific - pandemic, epidemic etc | Vaccination  Patient education or counselling - vaccination Collaboration - with wider teams involved in response | Increased accessibility to vaccination for rural and indigenous communities | Vaccine hesitancy and logistics in rural and remote areas |
| Ross B. et al. | 2014 | 97 | Case report (JCI) | Germany | HIC | Hospital | Not specified | Contamination of pharmaceutical product | Follow aseptic policy or guidance when making up pharmaceutical products - individual Follow aseptic policy or guidance when making up pharmaceutical products - organisation Pharmaceutical product source investigation |  | Delayed identification of the contaminated vials as the source of infection, difficulty in tracking the source across multiple hospital departments. |
| Routsolias J.C. et al. | 2020 | 62 | Expert opinion (JCI) | United States of America | HIC | Hospital | Pharmacist | Poisoning | Manage medicine and consumable supply issues - organisation Development of organisational policy or guidance Pharmaceutical care of patients Part of outbreak response Multi-Disciplinary Team Patient education and advice - treatment and follow up Long-term follow up and management of lasting effects of outbreak Collaboration at national level | Role of pharmacy professionals to overcome challenges faced by indigent patients, including limited access to medications, transportation issues, and lack of insurance coverage, which can affect treatment outcomes. | Limited availability of antidotes, challenges in patient compliance due to complex regimens, logistical issues related to patient follow-up and transportation |
| Rubin, S.E. et al. | 2014 | 63 | Expert opinion (JCI) | United States of America | HIC | Retail/community | Pharmacist | Influenza | Leadership role at national level |  |  |
| Ryan P. et al. | 2017 | 98 | Quantitative descriptive (MMAT) | Ireland | HIC | Hospital | Pharmacist | Clostridium difficile | Part of Infection, Prevention, and Control Multi-disciplinary Team |  | A significant portion of CDI-associated costs are fixed, limiting the potential savings from infection prevention efforts. The study highlights the importance of distinguishing between fixed and variable costs to avoid overstating the financial benefits of infection prevention. Investing in an antimicrobial pharmacist could be cost-effective if a sufficient number of CDI cases are prevented annually |
| Said M. et al. | 2023 | 171 | Quantitative descriptive (MMAT) | South Africa | LMIC | Hospital | Not specified | Contamination of pharmaceutical product | Recall of contaminated pharmaceutical products |  |  |
| Saleh E.A. et al. | 2022 | 160 | Quantitative descriptive (MMAT) | Yemen | LMIC | Retail/community | Pharmacist | Not specific - pandemic, epidemic etc | Supply of treatment to patients (OTC, sales or dispensing) Logistics and distribution of medicines or consumables - regional/organisational Manage medicine and consumable supply issues - organisation First point of patient contact | Pharmacists serving as frontline healthcare providers in the absence of functioning health institutions, allowing accessible healthcare to those in a war zone | Increase in the demand for prescription drugs without a prescription, lack of ongoing professional development. |
| Sallam, M. et al. | 2022 | 156 | Quantitative descriptive (MMAT) | Jordan | LMIC | Not specified | Pharmacy student | Mpox or Monkeypox | Knowledge about the disease/outbreak Undertake self education - Reduce misinformation |  | Barriers: The study points out that many students held conspiracy beliefs about the outbreak, indicating a gap in understanding that could hinder proper responses. |
| Santos-Pinto C.B. et al. | 2021 | 77 | Qualitative study (MMAT) | Brazil | LMIC | Primary care | Pharmacist | Zika | Patient education or counselling - prevention Pharmaceutical care of patients Part of outbreak response Multi-Disciplinary Team |  | Pharmacists had knowledge gaps regarding signs, symptoms, diagnosis, and epidemiological surveillance of Zika virus, limited training in public health among pharmacists, involvement of pharmacists in the epidemic response was minimal and often restricted to counselling. |
| Sar S. et al. | 2017 | 96 | Expert opinion (JCI) | Turkey | LMIC | Multiple | Pharmacist | Biohazard | Logistics and distribution of medicines or consumables - regional/organisational Supply of treatment to patients (OTC, sales or dispensing) Public education - reduce panic and fear Educate Health Care Workers about the disease Provide First Aid Patient education and advice Pharmaceutical care of patients Public education - reduce misinformation Stock management of medicines and consumables - organisation Maintain accurate and complete patient records. Development of organisational policy or guidance Stock management of medicines and consumables - organisation Development of national treatment policy, recommendations or guidance Disease surveillance -recognition and reporting of unusual medication requests Patient education or counselling - prevention Vaccination Supply preventative medicines or measures to patients - antibiotic prophylaxis |  | Need to be involved in policy planning, need to have education and training, ensure multi-disciplinary team working |
| Schwerzmann, J. et al. | 2017 | 64 | Quantitative descriptive (MMAT) | United States of America | HIC | Retail/community | Pharmacist | Influenza | Vaccination National public health program planning and implementation | Highlights the increased accessibility pharmacies provide compared to other providers (availability during off-hours), which could have indirect benefits in reaching underserved populations. | Variability in state practice laws and reimbursement processes limit the full utilization of pharmacies for vaccination. There is also limited public health program allocation to pharmacies. |
| Sharma, R. et al. | 2018 | 175 | Qualitative study (MMAT) | India | LMIC | Hospital | Not specified | Dengue | Stock management of medicines and consumables - organisation |  |  |
| Shehab N. et al. | 2018 | 65 | Qualitative study (MMAT) | United States of America | HIC | Compounding pharmacy or centre | Not specified | Contamination of pharmaceutical product | Follow aseptic policy or guidance when making up pharmaceutical products - organisation |  |  |
| Silvaggio, J.L. et al. | 2014 | 66 | Quantitative descriptive (MMAT) | United States of America | HIC | Retail/community | Not specified | Pertussis | Patient education or counselling - vaccination Vaccination Recognition and assessment of high-risk patients requiring vaccination - liaison with local schools |  | Variability in standard practices regarding pertussis vaccination across different provider types, challenges in adapting to new state laws, and increased awareness requirements. |
| Singh T. et al. | 2020 | 67 | Quantitative descriptive (MMAT) | United States of America | HIC | Retail/community | Pharmacist | Measles | Vaccination Organisational involvement in national vaccination program National vaccination programme planning and implementation |  | States with limited pharmacist vaccination privileges missed opportunities to increase vaccination rates during outbreaks. |
| Small, K.W. et al. | 2014 | 68 | Quantitative descriptive (MMAT) | United States of America | HIC | Compounding pharmacy or centre | Not specified | Contamination of pharmaceutical product | Involvement in patient identification, notification, and contract tracing Collaboration - with wider teams involved in response |  |  |
| Smith B.L. et al. | 2019 | 69 | Quantitative descriptive (MMAT) | United States of America | HIC | Retail/community | Pharmacist | Human immunodeficiency virus (HIV) | Supply preventative medicines or measures to patients - HIV PREP Patient education or counselling - prevention Point-of-care testing | Accessible access to prevention, treatment and testing for HIV | Financial constraints, consumable availability, and regulatory considerations |
| Smith, M. et al. | 2021 | 92 | Qualitative study (MMAT) | United Kingdom | HIC | Retail/community | Not specified | Human immunodeficiency virus (HIV) | Supply preventative medicines or measures to patients - HIV PREP | Accessible access to prevention, treatment and testing for HIV | Low health literacy & risk awareness among key at-risk groups. Unpredictable lifestyles interfering with regular medication schedules. |
| Sokolow L.Z . et al. | 2018 | 70 | Quantitative descriptive (MMAT) | United States of America | HIC | Retail/community | Multiple | Influenza | Vaccination Supply of treatment to patients (OTC, sales or dispensing) | Increased accessibility to vaccination and treatment | Lack of space for vaccine administration, and issues with physical environment to maintain infection control measures. |
| SteelFisher, G.K. et al. | 2018 | 71 | Quantitative descriptive (MMAT) | United States of America | HIC | Retail/community | Pharmacist | Influenza | Logistics and distribution of medicines or consumables - regional/organisational Supply of treatment to patients (OTC, sales or dispensing) |  | Concerns about antiviral shortages, infection risk to themselves and their families, legal liability, and limited capacity for handling increased workload and coordination with public health systems. |
| Subiros M. et al. | 2019 | 81 | Quantitative descriptive (MMAT) | France | HIC | Retail/community | Pharmacist | Food or water contamination | Supply of treatment to patients (OTC, sales or dispensing) Symptom recognition and diagnosis Disease surveillance - use of pharmacy medicines sale data for early outbreak detection | Limited access to clean water and sanitation, exacerbated the health impact of the water scarcity, disproportionately affecting vulnerable populations - pharmacists can support through surveillance etc. | Challenges in data collection due to limited healthcare infrastructure. |
| Sudakov, V.A. et al. | 2022 | 104 | Qualitative study (MMAT) | Russia | HIC | Retail/community | Not specified | Not specific - pandemic, epidemic etc | First point of patient contact |  |  |
| Sugawara T. et al. | 2018 | 119 | Quantitative descriptive (MMAT) | Japan | HIC | Retail/community | Not specified | Not specific - infectious diseases | Disease surveillance - use of pharmacy medicines sale data for early outbreak detection Supply of treatment to patients (OTC, sales or dispensing) |  | The system's effectiveness depends on the participation rate of pharmacies and the timely reporting of prescription data |
| Sutt, A.R. et al. | 2019 | 72 | Qualitative study (MMAT) | United States of America | HIC | Retail/community | Pharmacist | Influenza | Vaccination Vaccine dispensing Patient education or counselling - vaccination |  |  |
| Talarek, E. et al. | 2021 | 94 | Quantitative descriptive (MMAT) | Poland | HIC | Retail/community | Not specified | Not specific - pandemic, epidemic etc | Vaccination | Increased accessibility to vaccination |  |
| Tapia, M.D. et al. | 2016 | 73 | Quantitative RCT (MMAT) | United States of America | HIC | Not specified | Not specified | Ebola virus | Vaccine clinical trials - organisational involvement |  |  |
| Tegeltija, S. et al. | 2022 | 141 | Other | Multiple | n/a | Retail/community | Pharmacist | Not specific - pandemic, epidemic etc | Supply of treatment to patients (OTC, sales or dispensing) | Consideration of novel mechanisms to deliver medicines to reduce impact on vulnerable populations during outbreak |  |
| Valeri, F.et al. | 2014 | 106 | Qualitative study (MMAT) | Switzerland | HIC | Retail/community | Pharmacist | Not specific - infectious diseases | Assessing vaccination status Patient education or counselling - vaccination |  |  |
| Vendrik K.E. et al. | 2022 | 101 | Quantitative descriptive (MMAT) | Netherlands | HIC | Retail/community | Pharmacist | Impetigo | Part of outbreak response Multi-Disciplinary Team |  |  |
| Verger, P. et al. | 2018 | 83 | Quantitative Non-randomised (MMAT) | France | HIC | Retail/community | Not specified | Influenza | Vaccination | Increased accessibility to vaccination | Many at-risk individuals did not receive free vaccinations due to various administrative barriers (e.g., missed eligibility for vouchers, complex vaccination pathways). |
| Vicary, D. et al. | 2020 | 125 | Qualitative study (MMAT) | New Zealand | HIC | Multiple | Multiple | Food or water contamination | Disease surveillance - reporting of cases Public education Part of outbreak response Multi-Disciplinary Team Leadership role at organisational level Stock management of medicines and consumables - organisation Pharmaceutical care of patients Supply of treatment to patients (OTC, sales or dispensing) |  | Need for further research into the effectiveness of community pharmacists in public health surveillance. |
| Vila-Parrish, A.R. et al. | 2015 | 74 | Quantitative Non-randomised (MMAT) | United States of America | HIC | Hospital | Not specified | Influenza | Stock management and distribution of treatments, preventative measures or consumables - organisation |  |  |
| Vuillet-A-Ciles, H. et al. | 2015 | 82 | Narrative (JCI) | France | HIC | Retail/community | Not specified | Ebola virus | Public education Patient education and advice - disease Knowledge about the disease/outbreak Symptom recognition and diagnosis |  |  |
| Wong Y.J. et al. | 2022 | 122 | Mixed methods (MMAT) | Malaysia | LMIC | Retail/community | Pharmacist | Tuberculosis | Supply of treatment to patients (OTC, sales or dispensing) Adherence support for treatment Public education |  | Lack of confidence, insufficient knowledge in infection management, infection control concerns and the complexity of setting up designated areas. |
| Wong, Y.J. et al. | 2023 | 142 | Systematic review (JCI) | Multiple | n/a | Retail/community | Pharmacist | Tuberculosis | Supply of treatment to patients (OTC, sales or dispensing) Adherence support for treatment | Use of pharmacies to bridge care gaps and support treatment | Community pharmacists are underutilized in TB programs despite their potential contributions. |
| Zhang, C. et al. | 2017 | 115 | Quantitative descriptive (MMAT) | China | LMIC | Retail/community | Not specified | Influenza | Logistics and distribution of medicines or consumables - regional/organisational | Pharmacist role in ensuring fairness and access to medicines | Focus on balancing equitable drug distribution and accessibility. |
| Zhang, L. et al. | 2020 | 116 | Quantitative Non-randomised (MMAT) | China | LMIC | Not specified | Not specified | Influenza | Research - organisational involvement |  | Challenges include the potential biases in data (e.g., stockpiling of OTC medications, media-driven search query spikes), limitations of school-based ILI surveillance during non-school days, and the need to account for non-influenza respiratory pathogens |
| Zhao, Y. et al. | 2021 | 75 | Quantitative Non-randomised (MMAT) | United States of America | HIC | Retail/community | Multiple | Not specific - pandemic, epidemic etc | Logistics and distribution of medicines or consumables - regional/organisational Vaccination Undertake Self education - emergency response training National vaccination programme planning and implementation Organisational involvement in national vaccination program Supply of treatment to patients (OTC, sales or dispensing) |  | Limited actual participation in emergency response activities and training among pharmacists, low awareness and establishment of joint policies with health departments. |

S5. Risk of Bias Assessment. Y – Yes, N – No, NA – Not applicable, U – Unclear.

| Category | Question |
| --- | --- |
| **MMAT SCREENING QUESTIONS** | S1. Are there clear research questions? |
|  | S2. Do the collected data allow to address the research questions? |
| **1. MMAT QUALITATIVE STUDIES** | 1.1. Is the qualitative approach appropriate to answer the research question? |
|  | 1.2. Are the qualitative data collection methods adequate to address the research question? |
|  | 1.3. Are the findings adequately derived from the data? |
|  | 1.4. Is the interpretation of results sufficiently substantiated by data? |
|  | 1.5. Is there coherence between qualitative data sources, collection, analysis and interpretation? |
| **2. MMAT RANDOMIZED CONTROLLED TRIALS** | 2.1. Is randomization appropriately performed? |
|  | 2.2. Are the groups comparable at baseline? |
|  | 2.3. Are there complete outcome data? |
|  | 2.4. Are outcome assessors blinded to the intervention provided? |
|  | 2.5 Did the participants adhere to the assigned intervention? |
| **3.MMAT NON-RANDOMIZED STUDIES** | 3.1. Are the participants representative of the target population? |
|  | 3.2. Are measurements appropriate regarding both the outcome and intervention (or exposure)? |
|  | 3.3. Are there complete outcome data? |
|  | 3.4. Are the confounders accounted for in the design and analysis? |
|  | 3.5. During the study period, is the intervention administered (or exposure occurred) as intended? |
| **4. MMAT QUANTITATIVE DESCRIPTIVE STUDIES** | 4.1. Is the sampling strategy relevant to address the research question? |
|  | 4.2. Is the sample representative of the target population? |
|  | 4.3. Are the measurements appropriate? |
|  | 4.4. Is the risk of nonresponse bias low? |
|  | 4.5. Is the statistical analysis appropriate to answer the research question? |
| **5. MMAT MIXED METHODS STUDIES** | 5.1. Is there an adequate rationale for using a mixed methods design to address the research question? |
|  | 5.2. Are the different components of the study effectively integrated to answer the research question? |
|  | 5.3. Are the outputs of the integration of qualitative and quantitative components adequately interpreted? |
|  | 5.4. Are divergences and inconsistencies between quantitative and qualitative results adequately addressed? |
|  | 5.5. Do the different components of the study adhere to the quality criteria of each tradition of the methods involved? |
| **6. JBI Case reports** | 6.1. Were patient’s demographic characteristics clearly described? |
|  | 6.2. Was the patient’s history clearly described and presented as a timeline? |
|  | 6.3. Was the current clinical condition of the patient on presentation clearly described? |
|  | 6.4. Were diagnostic tests or assessment methods and the results clearly described? |
|  | 6.5. Was the intervention(s) or treatment procedure(s) clearly described? |
|  | 6.6. Was the post-intervention clinical condition clearly described? |
|  | 6.7. Were adverse events (harms) or unanticipated events identified and described? |
|  | 6.8. Does the case report provide takeaway lessons? |
| **7. JBI Narrative** | 7.1. Is the generator of the narrative a credible or appropriate source? |
|  | 7.2. Is the relationship between the text and its context explained? (where, when, who with, how) |
|  | 7.3. Does the narrative present the events using a logical sequence so the reader or listener can understand how it unfolds? |
|  | 7.4. Do you, as reader or listener of the narrative, arrive at similar conclusions to those drawn by the narrator? |
|  | 7.5. Do the conclusions flow from the narrative account? |
|  | 7.6. Do you consider this account to be a narrative? |
| **8. JBI Expert opinion** | 8.1. Is the source of the opinion clearly identified? |
|  | 8.2. Does the source of opinion have standing in the field of expertise? |
|  | 8.3. Are the interests of the relevant population the central focus of the opinion? |
|  | 8.4. Does the opinion demonstrate a logically defended argument to support the conclusions drawn? |
|  | 8.5. Is there reference to the extant literature? |
|  | 8.6. Is any incongruence with the literature/sources logically defended? |
| **9. JBI Policy/consensus guidelines** | 9.1. Are the developers of the policy/ consensus guideline (and any allegiences/affiliations) clearly identified? |
|  | 9.2. Do the developers of the policy/ consensus guideline have standing in the field of expertise? |
|  | 9.3. Are appropriate stakeholders involved in developing the policy/guideline and do the conclusions drawn represent the views of their intended users? |
|  | 9.4. Are biases due to competing interests acknowledged and responded to? |
|  | 9.5. Are the processes of gathering and summarizing the evidence described? |
|  | 9.6. Is any incongruence with the extant literature/evidence logically defended? |
|  | 9.7. Are the methods used to develop recommendations described? |
| **10. JBI Systematic review** | 10.1. Is the review question clearly and explicitly stated? |
|  | 10.2. Were the inclusion criteria appropriate for the review question? |
|  | 10.3. Was the search strategy appropriate? |
|  | 10.4. Were the sources and resources used to search for studies adequate? |
|  | 10.5. Were the criteria for appraising studies appropriate? |
|  | 10.6. Was critical appraisal conducted by two or more reviewers independently? |
|  | 10.7. Were there methods to minimize errors in data extraction? |
|  | 10.8. Were the methods used to combine studies appropriate? |
|  | 10.9. Was the likelihood of publication bias assessed? |
|  | 10.10. Were recommendations for policy and/or practice supported by the reported data? |
|  | 10.11. Were the specific directives for new research appropriate? |
